# Variation in shoot architecture traits and their relationship to canopy coverage and light interception in soybean (*Glycine max*)

**DOI:** 10.1186/s12870-024-04859-2

**Published:** 2024-03-16

**Authors:** Suma Sreekanta, Allison Haaning, Austin Dobbels, Riley O’Neill, Anna Hofstad, Kamaldeep Virdi, Fumiaki Katagiri, Robert M. Stupar, Gary J. Muehlbauer, Aaron J. Lorenz

**Affiliations:** 1 Department of Agronomy and Plant Genetics, University of Minnesota, 55108 St. Paul, MN USA; 2 School of Mathematics, University of Minnesota, 55455 Minneapolis, MN USA; 3 Department of Plant and Microbial Biology and Microbial and Plant Genomics Institute, University of Minnesota, 55108 St Paul, MN USA

**Keywords:** Canopy coverage, Shoot architecture, Light interception, *Glycine max*

## Abstract

**Background:**

In soybeans, faster canopy coverage (CC) is a highly desirable trait but a fully covered canopy is unfavorable to light interception at lower levels in the canopy with most of the incident radiation intercepted at the top of the canopy. Shoot architecture that influences CC is well studied in crops such as maize and wheat, and altering architectural traits has resulted in enhanced yield. However, in soybeans the study of shoot architecture has not been as extensive.

**Results:**

This study revealed significant differences in CC among the selected soybean accessions. The rate of CC was found to decrease at the beginning of the reproductive stage (R1) followed by an increase during the R2-R3 stages. Most of the accessions in the study achieved maximum rate of CC between R2-R3 stages. We measured Light interception (LI), defined here as the ratio of Photosynthetically Active Radiation (PAR) transmitted through the canopy to the incoming PAR or the radiation above the canopy. LI was found to be significantly correlated with CC parameters, highlighting the relationship between canopy structure and light interception. The study also explored the impact of plant shape on LI and CO_2_ assimilation. Plant shape was characterized into distinct quantifiable parameters and by modeling the impact of plant shape on LI and CO_2_ assimilation, we found that plants with broad and flat shapes at the top maybe more photosynthetically efficient at low light levels, while conical shapes were likely more advantageous when light was abundant. Shoot architecture of plants in this study was described in terms of whole plant, branching and leaf-related traits. There was significant variation for the shoot architecture traits between different accessions, displaying high reliability. We found that that several shoot architecture traits such as plant height, and leaf and internode-related traits strongly influenced CC and LI.

**Conclusion:**

In conclusion, this study provides insight into the relationship between soybean shoot architecture, canopy coverage, and light interception. It demonstrates that novel shoot architecture traits we have defined here are genetically variable, impact CC and LI and contribute to our understanding of soybean morphology. Correlations between different architecture traits, CC and LI suggest that it is possible to optimize soybean growth without compromising on light transmission within the soybean canopy. In addition, the study underscores the utility of integrating low-cost 2D phenotyping as a practical and cost-effective alternative to more time-intensive 3D or high-tech low-throughput methods. This approach offers a feasible means of studying basic shoot architecture traits at the field level, facilitating a broader and efficient assessment of plant morphology.

**Supplementary Information:**

The online version contains supplementary material available at 10.1186/s12870-024-04859-2.

## Background

Shoot architecture is defined by the shape, number and arrangement of individual phytomers that are the building blocks of a plant at any given period of time [1]. The modular nature of plants, resulting from repeating phytomer units, allows for complex structures to develop in response to changing environmental conditions and to accommodate growth requirements. While such modular development allows for a high degree of morphological plasticity across environments, a strong genetic component also underlies overall shoot architecture [2, 3].

Crop yields have shown steady and significant increases over the past several decades through the development of modern cultivars and advances in management strategies. Altering shoot architecture traits in wheat and rice through the introduction of semi-dwarf genes has contributed to dramatically increased yield when paired with more intensive management practices [4–6]. A reduction in plant stature was also accompanied by increased tillering and reduced lodging, which are also agronomically favorable. Subsequent studies showed that modifications in the genes governing the synthesis and regulation of gibberellic acid (GA) led to the development of semi-dwarf phenotypes, characterized by increased tillering and decreased lodging [4, 7]. In maize, more upright leaves which allow for more light penetration into lower parts of the canopy have accompanied selection for grain yield under increasingly high plant densities [8, 9]. Likewise, reduced tiller angle also allowed for increased plant density and consequently increased grain yield per unit area in rice [6, 8]. Because photosynthesis responds in a non-linear fashion to increasing light intensity [10], the top portions of the canopy may reach light saturation. On the other hand, lower parts of the canopy may experience a dearth in light intensity. Manipulation of leaf angles at different parts of the canopy (more vertical leaves in upper portion, more horizontal leaves in lower portion) could result in more even light distribution [10, 11].

Since 1940s soybean breeders have selected intensively for seed yield and harvestability traits such as lodging and shattering resistance [12]. Use of a relatively small number of cultivars for breeding, combined with intense selection for yield for many decades, has led to a narrow genetic base and limited variation of modern soybean adapted to North America [13, 14]. This limited genetic variation is apparent in the lack of diversity in canopy architecture among modern soybean cultivars. Moreover, it has been shown that the predominant canopy architecture of soybean is sub-optimal, with soybean plants possibly producing more leaves than needed [15]. A fully closed soybean canopy intercepts all the incoming light in the upper portions of the canopy, leaving these leaves saturated, while the lower leaves are shaded and underutilized [16–18]. Nevertheless, several architecture traits have been identified as contributing to increased yield potential including plant height, first pod insertion height, number of branches, internode length, stem diameter, number of nodes and leaf area index [19]. It has also been shown that certain architectural traits have a strong influence on light interception (LI) and canopy coverage (CC) in soybean [20, 21].

While both LI and CC are community properties wherein their effect influences soybeans at the stand or plot levels, they are influenced in turn by the shoot architecture of individual traits in the community. As such, many canopy architecture studies focus on the traits of individual plant shoot architecture. Attempts to measure shoot architecture have been either qualitative or limited to quantitative measurements of a few specific traits. In soybean, measurements of shoot architecture traits such as petiole length [22], branch number [23, 24], leaf width [25], petiole angle [26] and branch angle [21, 27] have shed light on genetic determinants of these traits. Studies attempting to relate shoot architecture traits to yield have been mixed [28, 29]. More recently QTL controlling branch angle in soybean was mapped to chromosome 19 [21, 27], a locus coincident to a previously mapped QTL for CC [30]. In addition, a leaf shape QTL has also been mapped to a position overlapping a CC QTL on chromosome 4 [21].

Early CC is essential for greater biomass accumulation and an important driver of yield [31] perhaps by improving water-use efficiency, maximizing LI, and weed suppression [32–34]. A few studies have identified QTLs for CC [21, 27, 30, 35], but the genetic control of shoot architecture traits that underlie variation in CC has not yet been thoroughly studied.

LI within fully covered canopies tend to be poor with less than 10% of light reaching lower levels of canopies [10]. Therefore, faster canopy coverage will likely have unintended consequence of suboptimal LI into the interior of the canopy. To obtain optimal CC without limiting LI in soybean canopies, a concept described as “smart canopies” [36], will require a comprehensive understanding of the architectural traits. Further, simple 2D imaging techniques that can be employed to analyze shoot architecture traits can be of value to obtain quantitative measure on otherwise intractable traits and their influence on CC and LI. The objective of this study was to identify, define and quantify soybean shoot architecture traits within a diverse set of accessions and examine their influence on both CC and LI.

## Results

### Rate of change in canopy coverage between vegetative and reproductive stage is different

**Fig. 1:**
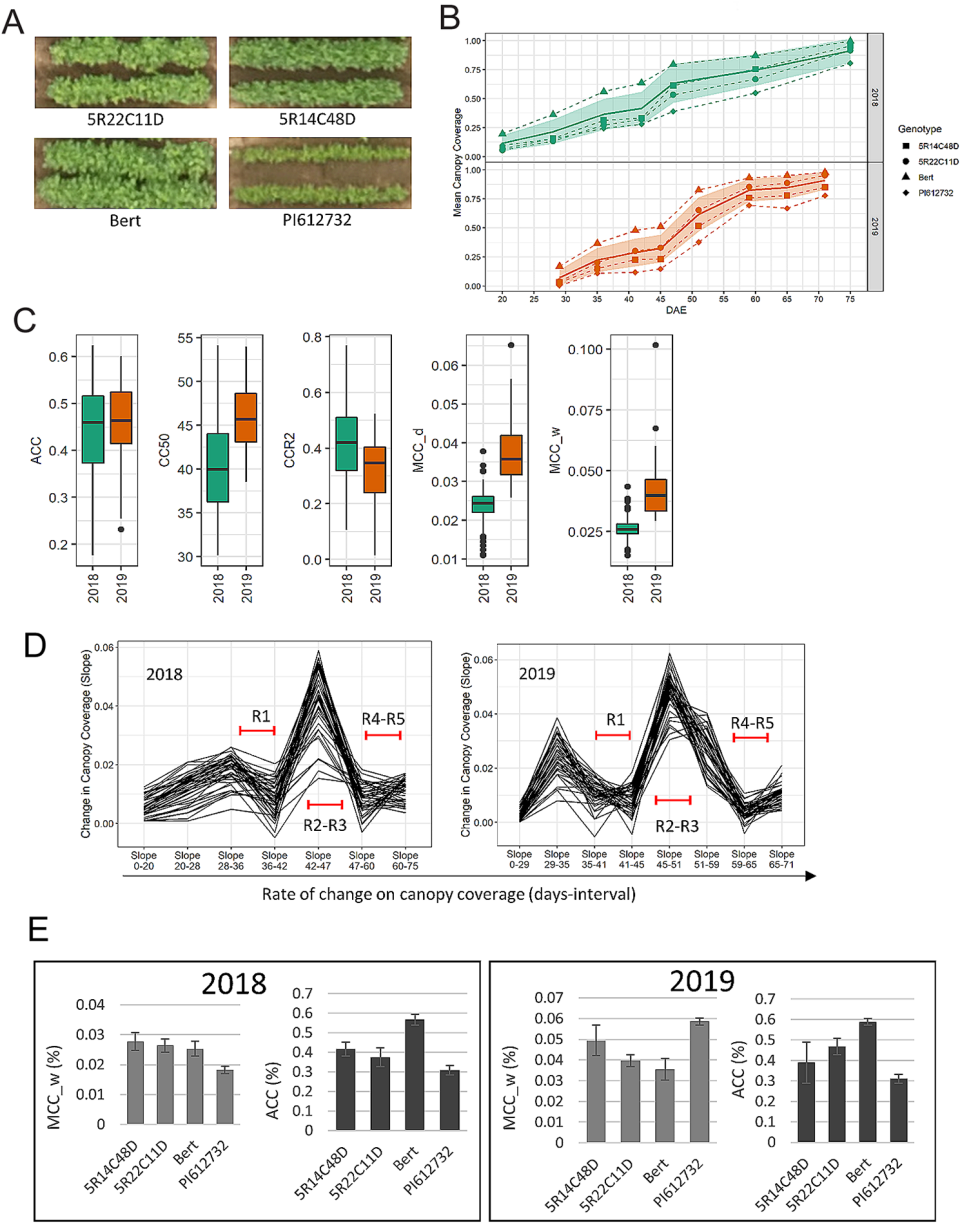
Variation in Canopy coverage between the different accessions. (**a**) Visual differences in canopy coverage between select accessions from UAV images (lower panels). (**b**) Canopy coverage over days after emergence (DAE) of select accessions. Mean canopy coverage of all the accessions in the study shown as shaded area. (**c**) Box plot showing the variation and range for different canopy coverage (CC) traits. Average Canopy coverage (ACC), days to 50% canopy coverage (CC50), canopy coverage at R2, Maximum daily rate of canopy coverage (MCC_d), and Maximum weekly rate of canopy coverage (MCC_w). (**d**) Rate of change in canopy coverage measured as slope between each measurement day with reproductive stages R1 to R5 indicated with red arrows. (**e**) ACC and MCC_w values for select accessions in 2018 and 2019

To explore variation in CC, we selected a panel of 40 soybean (*Glycine max*) accessions that displayed visual differences in shoot architecture (Table S1). There were large differences in CC between accessions easily visible from very early stages (Fig. 1a), with some reaching higher CC by R2 (full bloom) while several accessions never fully covered the canopy even by the end of the season (Fig S1; Table S2). When we examined time to 50% CC (CC50), a measure that allows us to examine CC especially during the early part of development, we found that some accessions reached CC50 as early as 30 DAE while others had CC50 values of over 50 DAE, a variation reflected in their average canopy coverage (ACC) over time (Table S2). The accessions with higher CC50 values outpaced CC from the start compared to those with lower CC50 (Fig. 1b, Table S2) There was substantial variation between accessions for CC-related traits such as ACC, CC50 and CCR2 (Canopy coverage at R2 or full flowering stage) (Fig. 1c; S2 and Table S3) validating our choice of accessions in this study.

To understand the magnitude of change in CC over time, we estimated the rate of change in CC between successive days on which CC was measured. There is a distinct reduction in the rate of CC starting at the beginning of the R1 stage and extending through the stages of appearance of first flowers. This is followed by a marked increase in the rate of CC beginning around the R2 stage (Fig. 1d). Our results showed that the maximum rate of CC occurred during the R2- R3 stages (Fig. 1d). A second decline in the rate of CC is seen between R4-R5 stages, coinciding with many accessions having reached their maximum CC. These results suggest that the rate of CC varies between the vegetative and reproductive stages with a boost in rate of CC following the transition of vegetative to reproductive growth.

While parameters such as ACC give a sense of variation in overall CC, they do not adequately convey at what stages accessions diverge from each other maximally or by how much they diverge from each other at their maximum rate of CC. To examine the variation in rate of CC between different accessions, we determined additional CC parameters from the logistic fit of the data, namely change in maximum rate of CC per day (MCC_d) and change in maximum rate of CC per week (MCC_w). MCC_w and MCC_d provide a glimpse of how fast the canopy is closing at the maximum rate of CC. We found that the rate of CC varies over the growing season. For example, in 2019 MCC_w values for Bert were less than PI612732 while the opposite was true in 2018. However, Bert clearly showed higher ACC in both years even though the rate of CC varied considerably between the accessions during the growing period. This suggests that a metric such as MCC_w can be useful in dissecting changes in CC rates during the season (Fig. 1e), potentially providing a fuller understanding of genetic variation in time to CC. We found that MCC_d, did not show significant correlation to any of our measured canopy parameters, suggesting the rate of change per day may not significantly impact the overall CC (Table S4). In conclusion, CC parameters can inform overall differences in canopy growth between different accessions, and can be used to express at which specific time point in soybean development variation can be observed.

#### Light interception is highly correlated with canopy coverage but only moderately with the rate of canopy coverage

**Fig. 2:**
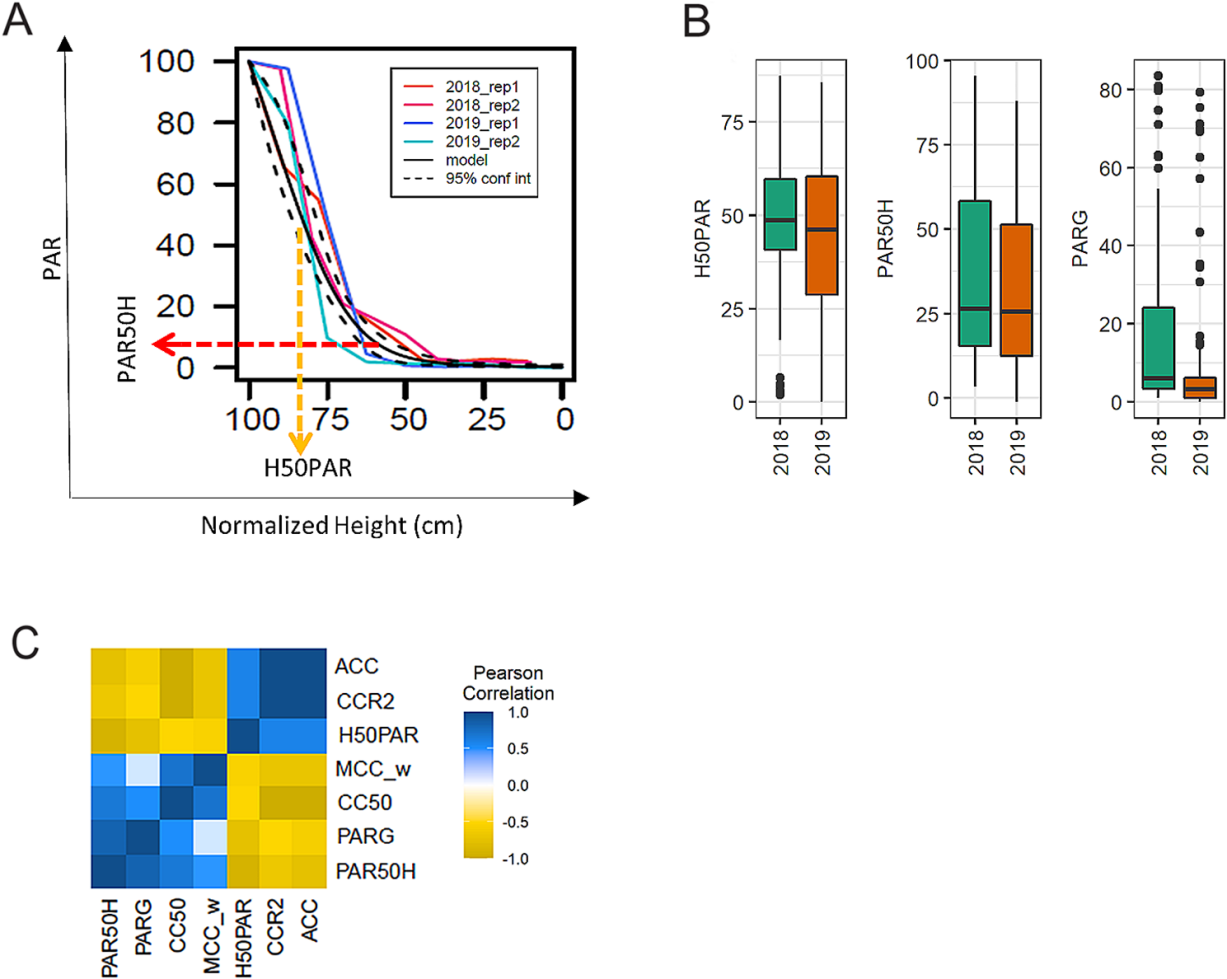
Light Interception (LI) within the soybean canopy can be modeled using logistic regression. (**a**) Photosynthetically active radiation (PAR) measured using a line bar at 10 cm increments from ground up and a logistic model fit to the data. Fit for one accession is shown as an example including two reps each from 2018 and 2019 (different colors) as well as the mean (black solid line) is indicated. 95% confidence interval for the fit is indicated (black dashed line). Light reaching the ground (PARG), height at 50% PAR (H50PAR) and PAR at 50% height (PAR50H) were calculated from the fit. (**b**) Variation for LI traits is shown in box plots. (**c**) Best linear unbiased predictions (BLUPs) were calculated from LI and CC data collected in 2018 and 2019 and used for correlation analysis. Pearson correlation (FDR adjusted p value < 0.05) between different traits is shown as a heat map

Previous studies have indicated that LI and CC are highly correlated [37]. A logistic model was determined to be a good fit to approximate LI in soybean canopies (Fig. 2a, S3a and b). PAR at 50% of plant height (PAR50H), height at which 50% PAR (H50PAR) is reached and PAR at ground level (PARG) were calculated from the model fit (Fig. 2a). These parameters were chosen as metrics for LI within the soybean canopy because we sought to understand how variation in shoot architecture and CC affects light environments within the canopy. As with CC parameters, the accessions in our panel showed a wide range in variation for LI parameters (Fig. 2b; S2). To understand how CC and LI within the canopy are related, we examined the correlation between the LI and CC parameters. We found that all three LI parameters were highly correlated with the CC parameters ACC, CC50, and CCR2. They were also significantly but moderately correlated with MCC_w, suggesting that LI is impacted overall by CC but to a lesser extent by rate of CC. LI parameters were not significantly correlated with MCC_d (Fig. 2c), suggesting that the rate of change in CC per day does not significantly impact LI. The two LI parameters H50PAR and PAR50H are negatively correlated with one another (Table S4). Accessions allowing for better light transmission within the canopy interior will have higher PAR50H and conversely, lower H50PAR since light can reach much lower into the canopy and the height at which 50% PAR is reached is closer to the ground.

#### Variation in plant shape is on a continuous scale and impacts light interception and CO_2_ assimilation

**Fig. 3:**
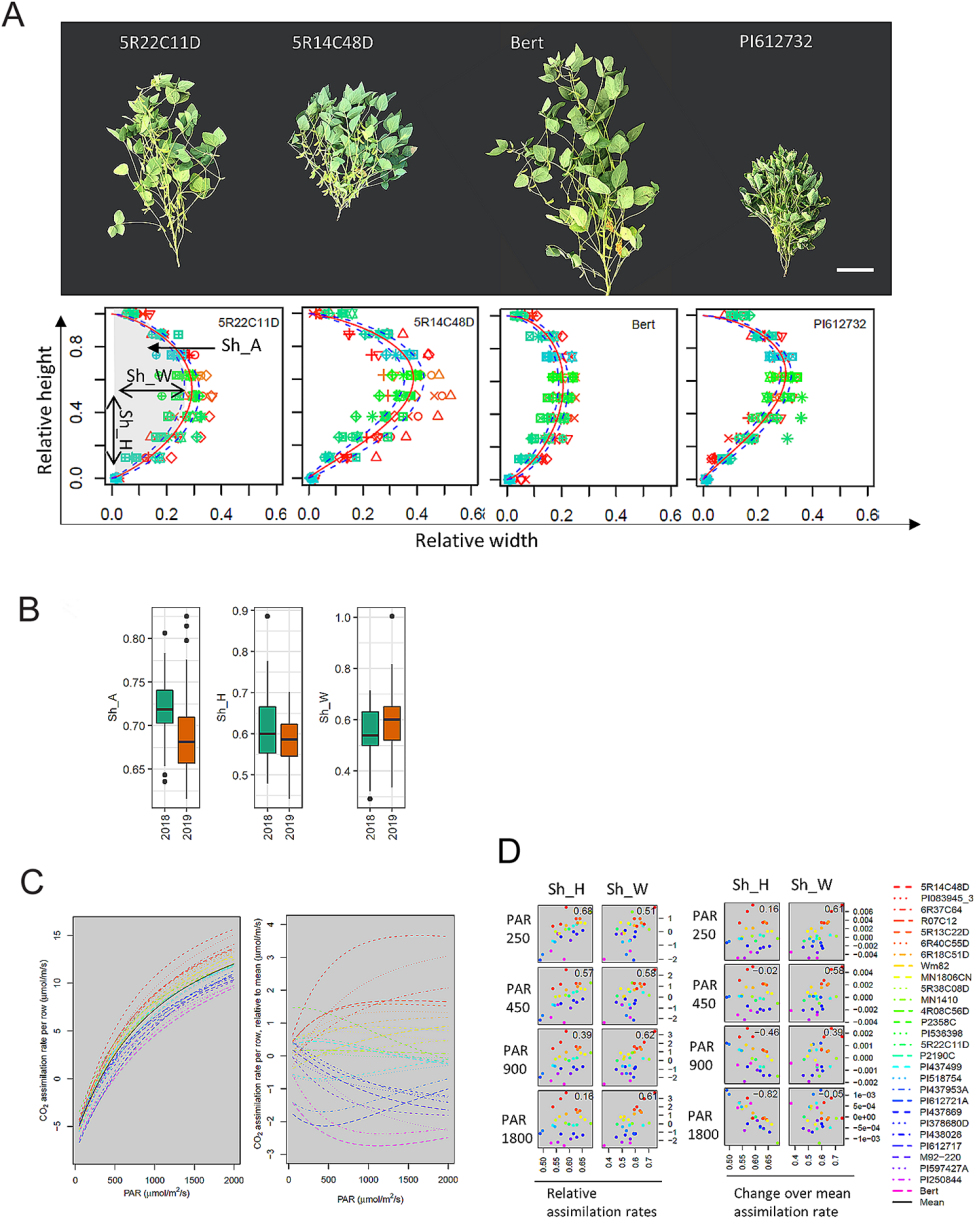
Variation in soybean shoot architecture at individual plant level described in terms of shape parameters. (**a**) Width of the plant along the length of the plant was measured at 0, 12.5 25 37.5, 50, 65.5, 75, 87.5 and 100% height of the plant. A beta distribution function was used to fit the width data to approximate the shape of the plant. Outcome of a beta distribution fit for four visually distinct accessions (upper panel) shown in the lower panel. From the fit, the overall shape could be described in terms of three parameters, peak height (Sh_H), area under the curve (Sh_A), and width scaling factor (Sh_W) (**b**) Variation for plant shape parameters from 2018 and 2019 is shown in box plots (**c**) CO_2_ assimilation rate plotted against the PAR levels in one-meter-long plant row where the plant shape is modeled using the beta-distribution. Different accessions are in different colors with the mean value in black (left panel). The differences in the CO_2_ assimilation rate between each accession from mean is shown (right panel). (**d**) Correlations between the relative assimilation rate and the two shape parameters (Sh_H and Sh_W) across the genotypes at four representative PAR values, 250, 450, 900, and 1800 µmol/m 2 /s shown (left panel). Change in relative assimilation can be observed as the slope of the relative assimilation rate at particular PAR level. Correlations between the slope of the relative assimilation and Sh_H or Sh_W at the four PAR levels are shown for each accession (right panel)

Variations in shoot architecture can manifest as variation in overall shape of the plants that in turn can impact the LI and CC. To compare plant shape, we sought to express shape in terms of comparable parameters. We measured plant shape by parameterizing the two-dimensional space occupied by the soybean plant. The width of individual plants was measured along the length of the plant and the shape was approximated by fitting a beta distribution model to the data. Three parameters were described: Sh_W approximates the widest part of the plant (maximum distance from the center line) Sh_H captures the height of the plant at which the largest width occurs and Sh_A quantifies the area under the curve (Fig. 3a, S4). The shape of each individual plant can thus be described using these three parameters that show high variation between the accessions in our panel (Fig. 3b; S2). Both SH_W and SH_A were found to be genetically determined, with reliability (${i}_{ACC}^{2}$) values of 0.7 and 0.6, respectively. However, Sh_H had a relatively lower ${i}_{ACC}^{2}$, being only 0.4, suggesting that non-genotypic effects are a relatively more important source of variation for this trait. Therefore, plant shape can be analyzed using simpler 2D imaging that easily captures the main and significant differences between accessions without the need to resort to lower-throughput 3D reconstruction methodologies.

Next, we sought to understand the influence of plant shape on light interception within the canopy. Previous studies have established the light response curve for soybean under various conditions using experimental as well as biophysical modeling [10, 38, 39]. Based on light response curve from [38] we predicted the CO_2_ assimilation values for different accessions in our study by calculating the assimilation rate at different PAR (Fig. 3c, left panel). To make comparison of the assimilation rate at a particular PAR value across the accessions easier, the relative assimilation rates of the accessions were subtracted from the mean (Fig. 3c, right panel). 5R14C48 and Bert had the highest and lowest relative assimilation rates, respectively, across PAR levels except for at very low PAR levels, perhaps a reflection of differences between their shapes (Fig. 3a).

Next, potential correlations between the relative assimilation rate and the two shape parameters (Sh_H, relative height of the widest part, and Sh_W, the maximum width) across the accessions at four representative PAR values, 250, 450, 900, and 1800 µmol/m2/s, were investigated. The relative assimilation rate was positively correlated with Sh_W across the PAR levels (Fig. 3d, left panel). The impact of Sh_W becomes apparent when examining the example of 5R14C48, which exhibits higher Sh_W values than Bert. This higher Sh_W is associated with increased PAR interception within the canopy and greater relative assimilation, as illustrated in the plots depicting the assimilation rate versus Sh_W across all four PAR levels. High Sh_W values translates to widest part of the plant towards the top of the plant (inverted cone shape) while low Sh_W values would mean the widest part of the plant is towards the bottom part of the plant (cone shaped). The relative assimilation rate is positively correlated with Sh_W at low PAR values, while almost no correlation was observed at the highest PAR values. The positive correlations at the low PAR values indicate that a flat-top shape is more photosynthetically efficient than a cone shape at low light intensity.

Next, we focused on how the relative assimilation rate changes when the PAR increases. This relative assimilation change can be observed as the slope of the relative assimilation rate at particular PAR level. When we look at the correlations between the slope of the relative assimilation and Sh_H or Sh_W at the four PAR levels, we observe that the slope is highly negatively correlated with Sh_H at the highest PAR level suggesting that a more conical shape at the top is likely more beneficial to harness the excess light allowing for more light penetration into the canopy (Fig. 3d right panel). Therefore, plant shape exerts influence on light interception and rate of CO_2_ assimilation at various levels of plant canopy.

#### Variation in soybean canopy properties can be described in terms of individual quantifiable and genetically variable shoot architecture traits

**Fig. 4:**
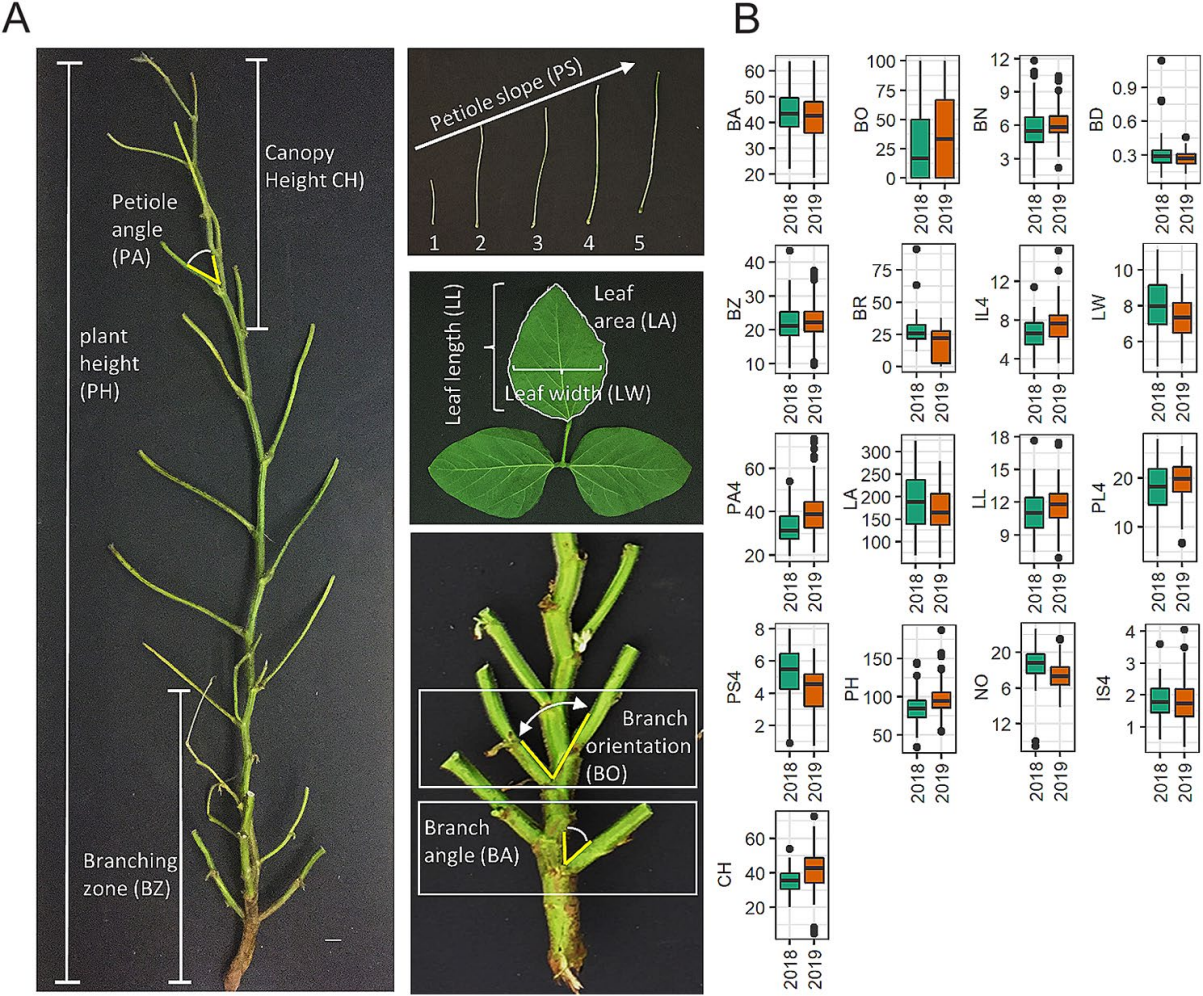
Shoot architecture of Soybean can be described by individual traits. (**a**) Plants were defoliated and branches trimmed before imaging. Different traits that were measured from the images are shown. (b) Variation for each trait is shown as box plots. Traits included are Branch angle (BA), Branch orientation (BO), Branch number (BN), Branch density (BD), Branching Zone (BZ), Branch Ratio (BR), Internode length at node 4 from the top of the plant (IL4), Leaf Width (LW), Petiole angle at node 4 from top of the plant (PA4), Leaf area (LA), Leaf Length (LL), Petiole length at node 4 from the top of the plant (PL4), Slope of top four petioles (PS4), Plant Height (PH), Number of nodes (NO), Slope of the top four internodes (IS4), and Canopy height (CH)

While some traits that describe soybean shoot architecture have been described and made available as soybean structural ontologies (SOYBASE: https://www.soybase.org/ontology.php), this study describes novel traits, measurement criteria and motivation for introducing these traits. To gain a more comprehensive insight into shoot architecture traits, we employed destructive sampling of plants except for petiole angle that was measured non destructively, directly on the plants. Non-destructive sampling in soybeans at full canopy coverage proves challenging, as many of the traits are concealed within the foliage. (For details on how specific measurements were done see Methods) As such, we describe soybean architecture traits in this study under four broad categories (1) branch-related traits; (2) leaf-related traits; (3) whole-plant related traits; and (4) traits related to the top of the plant (Fig. 4a; Table S5). In soybeans the top 25% of the canopy absorb the majority of incoming radiation [40–42]. Indeed, from our visual inspection, during the R2 stage, when CC rate is maximum, much of the visible canopy that the incident light encounters is composed of the top 5–6 nodes and hence held more relevance. Therefore we decided to focus on shoot architecture traits that can be delimited to this theoretical zone of maximum solar radiation exposure. Accordingly, the fourth category of traits such as petiole length (PL), Petiole angle (PA) the internode length (IL), and Canopy height (CH) we chose to focus our measurements at the top of the plant.

In this study we introduced additional traits to assist in quantifying overall shape of the top of the canopy in terms of “slope of internode length” (IS) and “slope of petiole length” (PS) (Fig. 4a; Fig S6a and S6b). We found that plants with nodes placed progressively further apart and thus with progressively longer internodes have a steeper incline and higher slope. However, plants displaying a more rounded shape at the top of the canopy will show a lower internode slope with increasingly longer petioles from the top (Fig S5b). The shape can also be influenced by the angle at which petioles are held from the main stem, measured here as petiole angle (PA). Thus, we found that shape of the plant at its apex can be parameterized using traits such as IS, PS, and PA.

Next, we wanted to understand how branches contribute to CC and LI by defining branching traits in more specific and measurable units, in a way that has not been done previously. Accordingly, the traits branch angle (BA) describe the angle at which the organs are held from the main stem. Distribution of branches was quantified in terms of the following four parameters: 1. Distance from the bottom of the plant to the last branch initiated, which we termed “branching zone” (BZ); 2. Average branch number per plant (BN); 3. Number of branches per unit length of the branching zone, termed “branching distribution/density” (BD); 4. Ratio of the branching zone to total plant height, termed “branching zone ratio” (BR), which quantifies the proportion of the stem that bears branches.

All of the traits we examined exhibited variation between accessions (Fig. 4b; S2 and Table S3). Reliability estimates were also found to be high for the shoot architecture traits (Table S3), suggesting they are under strong genetic control. Thus, we are able to describe shoot architecture in terms of quantifiable traits. Next, we decided to use the traits we quantified to examine how shoot architecture of individual plants relate to plot level traits such as CC and LI.

### Canopy coverage and light interception are influenced overlapping as well as independent shoot architecture traits

**Fig. 5:**
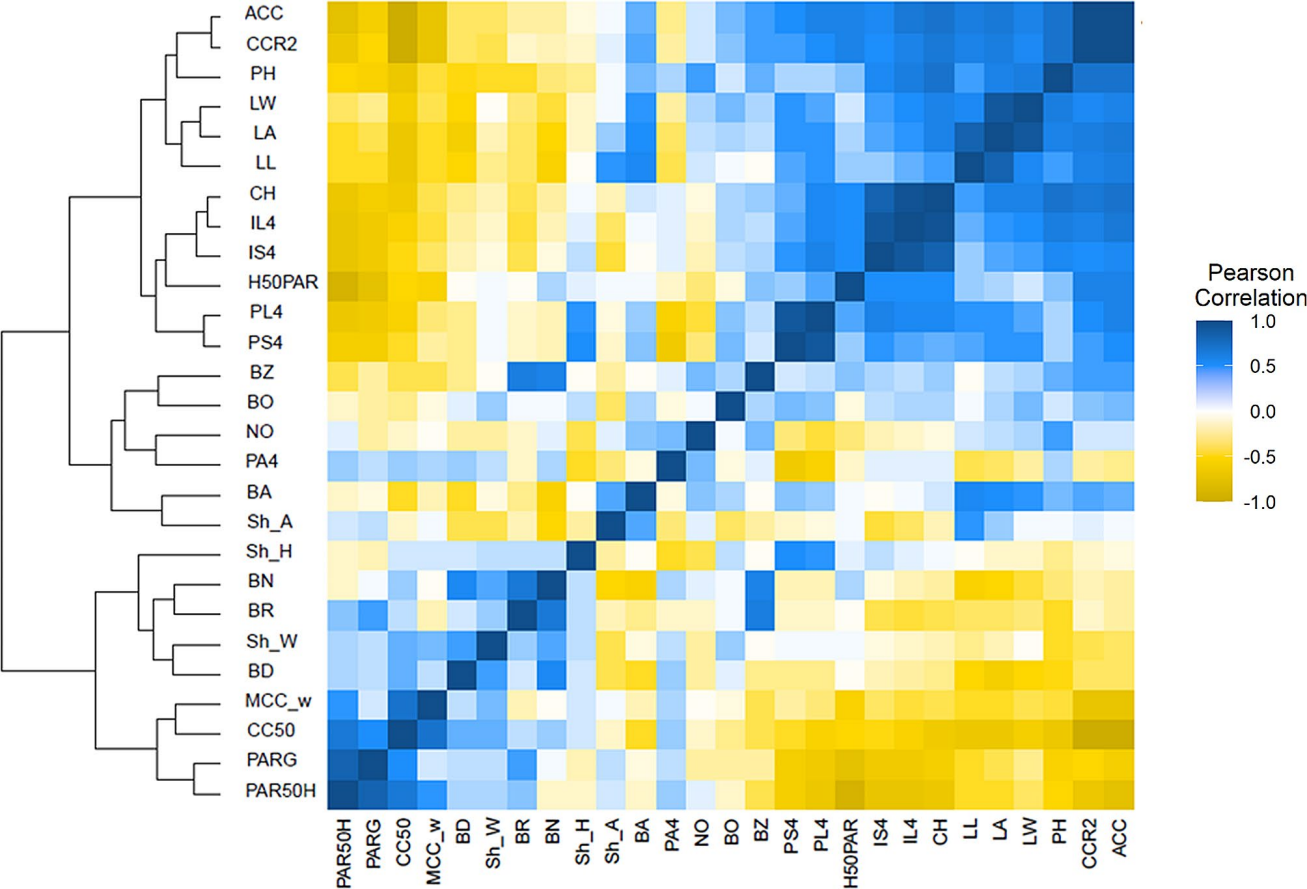
Shoot architecture traits show high correlation to both canopy coverage as well as light interception in soybean. Best linear unbiased predictions (BLUPs) were calculated from all 2018 and 2019 data and used for correlation analysis. Pearson correlation (FDR adjusted p value &lt; 0.05) between different traits is shown as a heat map. Traits in the heat map are: Branch angle (BA), Branch density/ distribution (BD), Branch number (BN), Branch orientation (BO), Branching zone (BZ), Branch ratio (BR), Days to 50% canopy coverage (CC50), Canopy coverage at R2 (CCR2), max growth rate (%/week) (MCC_w), Average Canopy Coverage (ACC), Canopy Height (CH), Height at 50% tPAR (H50PAR), tPAR at 50% height (PAR50H), tPAR at ground level (PARG), Internode length at node 4 from the top of the plant (IL4), Slope of the top four internodes (IS4), Leaf area (LA), Leaf Length (LL), Leaf Width (LW), Number of nodes (NO), Petiole angle at node 4 from the top of the plant (PA4), Petiole length at node 4 from the top of the plant (PL4), Slope of the top four petioles (PS4), Plant Height (PH), Plant shape parameter peak height (Sh_H), Plant shape parameter area under the curve (Sh_A), and Plant shape parameter width scaling factor (Sh_W)

Correlations between individual shoot architecture traits and CC and LI parameters were used to identify relationships between these traits. Best linear unbiased predictions (BLUPs) were calculated from all 2018 and 2019 data (Table S4) and used for correlation analysis. ACC was positively correlated with plant and canopy height (Fig. 5; Table S4). Additionally, ACC was positively correlated with leaf area, leaf length and leaf width, as well as internode length and petiole length. Predictably, leaf architecture traits negatively correlated with rate of CC (CC50 and MCC_w) (Fig. 5; Table S4), suggesting that leaf traits may drive the rate of CC to achieve faster CC. Plant height, canopy height, leaf traits, and internode and petiole lengths were significantly correlated to LI traits. Both petiole and internode length showed strong negative correlation with LI traits PARG and PAR50H (Fig. 5; Table S4) that are indicators of light penetration within the canopy. Similarly, leaf architecture traits also showed negative correlation to PAR50H and PARG, indicating that taller plants with larger leaves show faster CC at the cost of light penetration towards the interior of the canopy.

We examined the influence of branching traits on CC. We found that branch angle (BA) showed a negative correlation to CC50, but we could not detect a significant positive association between ACC and BA (Fig. 5; Table S4). Therefore, manipulation of branch angle may be an avenue to achieve faster CC but perhaps the advantage is more at the earlier part of growing season. The CC traits ACC and CCR2 showed positive correlation to the branching zone, indicating that accessions that had branches distributed further along the main axis showed higher CC.

We examined if BA or BZ influenced LI and found no significant correlation between these traits. Other branching traits such as branch number, orientation or branch ratio (BN, BO and BR) did not seem to be strongly correlated with CC or LI traits (Fig. 5; Table S4). Branching zone was positively correlated to number of nodes (NO) as well as plant height (PH), suggesting taller plants with higher number of nodes branched more extensively along the stem than shorter plants with fewer nodes. Although at lower canopy, CC and LI are negatively correlated, we found useful variation and correlations between CC, LI, and many shoot architecture traits that could possibly be leveraged to achieve a balance between time to CC, ACC and LI.

## Discussion

Earlier canopy coverage is associated with increased yield [30]. A denser canopy however has the unintended consequence of suboptimal LI into the canopy interior. To obtain optimal CC without limiting LI in soybean canopies, it is important to understand how individual shoot architecture traits manifest in variation for canopy-level traits of soybean. Toward this end, we identified and established protocols to quantify soybean shoot architecture traits to study their relationship with CC and LI. In row crop such as soybean, traits such as CC and LI are community properties having a collective impact on the plants. Similarly, the shoot architecture traits of individual plants in that community is consequential. Our study systematically and quantitatively examined individual plant traits in soybean and determined how these traits influenced canopy properties. In doing so, we have defined new traits and methods for their measurement. An important feature of this study is the fact that all the data was collected from field plots. Soybean displays high plasticity in its architectural forms, and therefore data collected in greenhouse and controlled growth chamber environments may not translate to field conditions.

### Rate of canopy coverage changes over the growth period with pronounced differences between specific developmental stages

Canopy coverage has previously been expressed in terms of ACC values from multiple seasons [30] and fractional CC representing the visible green pixel in a unit area [37]. While these terms sufficiently describe the overall or aggregate CC, we sought additional parameters that capture the trajectory of CC. In other words, while previous terms capture the gross differences in CC between accessions, it does not adequately capture the underlying causes for these differences at the individual plant level. Additional traits measured in this study, such as MCC_w and CC50, may help explain variation in the rate of CC during the growing season. For example, as the growing season progresses CC values can be distorted due to factors such as lodging. While we did not encounter significant lodging by the time of phenotyping, it is a conceivable source of variation later in the season. In addition, different soybean accessions may respond to environmental variations differently giving rise to localized variation in CC rates during the growth season and thus average CC may not be sufficient to quantify such variations [43]. Furthermore, the impact of changes in biomass accumulation (consequently CC) and yield may differ significantly based on the growth stage of the plants and such differences may vary between accessions [44]. Our study was limited to 40 accessions, but we envision that in larger studies including more accessions, we could use the new parameters we have defined to quantify variation in rate of CC to select soybeans that may respond favorably to adverse environmental conditions at key growth stages.

We found that the rate of CC is not uniform throughout the season. A rapid increase in CC after germination is followed by a brief reduction in rate of change CC, specifically before the appearance of the first flowers (Fig. 1d). In most plants, transition to flowering from the vegetative stages is marked by several important changes at a molecular, physiological and morphological levels [45]. The many changes that occur during floral transition are revealed during the initiation of floral organs and changes in leaf, stem, phyllotaxy and growth rate [45]. The timing of transition to flowering can be critical for seed setting and yield where a transition that occurs too early results in insufficient vegetative biomass accumulation, while a transition that occurs too late could result in excessive vegetative biomass accumulation to the detriment of yield [46]. A small reduction in rate of change in CC before floral initiation and seed set may indicate a brief adjustment in resource allocation from vegetative to reproductive stages and possibly nutrient partitioning at seed set. We found that the brief decline in growth rate observed before floral initiation was followed by a steep increase in growth rate. While we did not observe any significant environmental changes such as sudden temperature changes or water deficit during the floral transition during both years of our study (Table S6), other unknown environmental impacts that we have not accounted for cannot be ruled out. We also saw a reduction in the rate of CC between R4 and R5 stages, when the soybeans are beginning to fill seeds. A majority of accessions in our study have already closed the canopy by the R4 stage, therefore effectively appearing as a reduction in the rate of CC. In conclusion, a closer inspection of CC, especially during the floral transition with a larger number of accessions in multiple locations might shed further light on the subject.

### Individual architecture traits are key determinants of canopy coverage and light interception

Plant architecture is a primary determinant of LI as well as CC. We have deconstructed the shoot architecture of soybean into several individual measurable component traits to determine how they affect CC and LI. We found that leaf traits such as leaf area have a strong influence on CC. We also found that traits associated with the top of the soybean plant pertaining to the zone of maximum solar radiation exposure, show strong correlation to both CC as well as LI. In most accessions, the lower part of the canopy is obscured after the canopy is completely covered and the top of the plant is the only apparent part of the plant that can intercept light. Therefore, a canopy structure that can allow deeper light penetration is desirable. The 3D structure of the plant pertaining to the top few nodes and associated structures become important towards ensuring deeper penetration of light into the canopy. Accordingly, in our study we found that the shape and structure of the top portion of the plant can be modeled. We found that parameters that indicated the projection of the top of the plant above the canopy level can be useful indicators of effectiveness of light penetration into the canopy as evidenced by a strong correlation of LI with internode and petiole slope. Surprisingly however, petiole angle (measured non-destructively and directly on the plant) did not seem to be strongly associated with LI. Petiole angle in soybean shows a diurnal pattern and changes throughout the day and is strongly influenced by light [47, 48]. It is possible that our measurement methods were not timed appropriately to account for variation due to such changes and hence not an accurate representation of the actual variation in petiole angle between the accessions.

Branching traits associated with angle and distribution of branches along the main stem seemed to be of moderate consequence to CC. Branch number or orientation did not impact CC. As stated above, the foliage associated with the top part of the soybean obscures the lower parts of the plant. As such, the effect of branch-related traits to CC and LI becomes harder to delineate. If the number of leaves on the plant could be reduced, the arrangement of branches and the other branching traits described in our study may become more consequential in achieving a better display of photosynthetic surfaces, thus achieving a balance in CC, LI and photosynthesis. Evidence suggest that soybeans have excess foliage and reducing the overall leaf area may in fact increase yield [15]. An additional advantage of using branching traits to achieve faster CC and LI will be to increase the number of pod-bearing nodes available per plant to take advantage of better LI and presumably higher photosynthesis.

### An ideotype for canopy coverage and light interception in soybean canopies

**Fig. 6:**
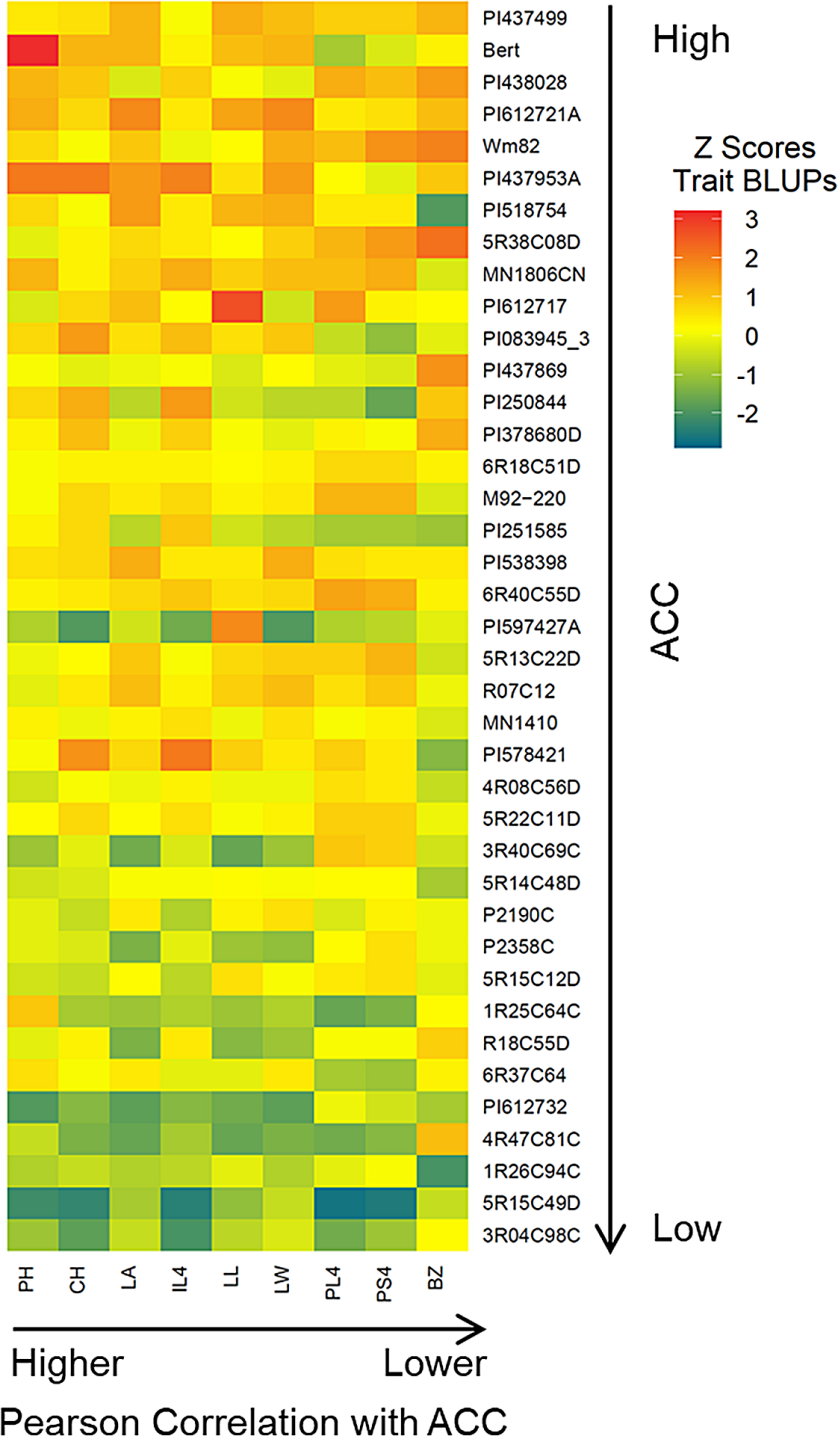
Plants with high canopy coverage are taller and have larger leaves with longer petioles. (**a**) Heatmap showing accessions ordered from top to bottom with higher to lower canopy coverage and traits ordered left to right with higher to lower correlation with average canopy coverage ACC

From this study, it is evident that shoot architecture traits have a significant influence on CC as well as LI within the soybean canopy. In our study, taller accessions with larger leaf area, length and width have faster CC with better LI at the top of the canopy. Consistent with this study, soybean with lanceolate leaf morphology with longer leaves has been related to increased LI [49]. Therefore, an ideotype for achieving high CC would be a plant that is tall with larger, longer leaves. While having higher values for these traits is ideal, some traits like PH can have a negative influence on yield by increasing chances of lodging [50]. However, it is possible to obtain similar results by varying other architecture traits. In essence, high values for one or more traits could compensate for traits that vary from the current model of tall plants with large leaves. For example, plants that are shorter could achieve higher CC by having a larger branching zone, e.g.: 5R38C08D. Similarly, an accession such as PI612717 can achieve high CC with longer lanceolate even with modest plant height (Fig. 6).

Previous studies have found a strong positive correlation between LI and CC [37]. Most of the light however is intercepted at the top of the canopy with lower light penetration into the lower portions of the canopy. By parameterizing LI at various levels within the soybean canopy we were able to show that plants with high CC tend to have limited light transmitted lower through the canopy. Our LI trait H50PAR that indicates height at which 50% radiation is intercepted is correlated positively with CC, suggesting most of the light is blocked by the top part of the canopy in these accessions. A trade-off between overall CC as well as rate of CC and light penetration into the lower levels of canopy was evident. Previous studies have shown that loss of photosynthetic efficiency due to shading from upper canopy can be reduced by better light penetration by more erect leaves in Sorghum bicolor [51]. In our study, CO_2_ assimilation modeled at different light penetration within the canopy suggests that similar advantage may be obtained in soybean plants with a more cone shaped upper canopy to allow for deeper light penetration. It is possible to realize such a plant shape by varying the node distribution (internode slope) and petiole lengths (petiole slope) at the top of the plant, an arrangement where each successive leaf from the top has progressively longer petioles spaced further below from each other. Such an arrangement may allow for better light penetration into the canopy while not affecting CC. Our model is based on the assumption of a specific solar angle at midday and therefore likely show differences at other solar angles. Additionally, our model focuses solely on architectural traits and how light interception influences CO_2_ assimilation. Other important factors such as leaf photosynthetic rates, nutrient availability, and other physiological factors are likely to have a significant effect on overall CO_2_ assimilation but does not fall under the purview of this study.

As discussed previously, plants with most of their branches distributed towards the lower part of the main stem, i.e. lower BZ values, would allow for deeper light penetration into the canopy without significantly reducing CC. A narrower angle of branching would further increase light penetration into the canopy, but this must be accompanied with consideration to CC. A balanced approach towards angle of branching and distribution of branches will be required to achieve optimal CC and LI. In addition, overall height of the plant can be higher along with larger lanceolate leaf area (allowing for better lower canopy light interception), both of which seem to be important for CC, but would not negatively impact LI. We also found several branch-related traits that could prove to be useful as independent factors that affect plant structure and hence are deserving of further examination, even though they were not significantly correlated with CC or LI in our study.

## Materials and methods

### Plant material and field experimental design

A list of the plant material used in the study is presented in the supporting material (Table S1). The accessions included in this study were a combination of accessions from the U.S. germplasm collection, released public cultivars, and selected mutations from the fast neutron mutant population [52]. For simplicity, we will use “accession” to describe the genotypic units The accessions were selected based similar phenologies (except WM82) and for variation in shoot architecture on visual inspection. All accessions were grown in two-row plots planted east-west, 3 m in length, spaced 0.76 m apart, and planted to a density of 15 seeds/m to allow for maximum expression of genetic variation in shoot architectural traits without significant impact from crowding. The trial was grown in St. Paul, MN in 2018 and 2019. The experimental design was a randomized complete block design with four replications in 2018 and two replications in 2019. Lodging was not observed at the time of shoot architecture measurements in either growing season.

### Imaging and data extraction

Plants were imaged in-field using a smartphone mounted on a tripod. At least six plants per field plot were imaged at the R2-R3 (full flowering to early pod-set) stage of development [53]. Petiole angle (PA) was imaged non-destructively in the field at the 4th and 5th node from the top of the plant by placing a black background and imaging the angle between the main stem and the petiole. Whole plants were manually defoliated and imaged by laying them horizontally on a black background. Petioles from the top seven nodes were imaged for each plant. Fully expanded leaves from the middle of each plant were harvested to image leaf shape and area. ImageJ (https://imagej.nih.gov/ij/; [54] open-source image analysis software was used to measure the branch angle, petiole length, internode length, leaf length and leaf width. Leaf traits were measured for each leaflet and mean values reported.

To quantify the steepness of the top portion of the plant, petiole length was plotted against a numeral designation of the node from which the petiole was sampled, where the uppermost node was designated as node number one, the second uppermost node was designated as node number two, and so on. A linear regression coefficient was estimated from a simple linear regression model and referred to as “petiole slope” (PS4). The length of the internode below each node numbered as described above was also measured and similarly fitted to a linear regression model to estimate “internode slope” (IS4). Canopy height (CH) was defined as the sum of the length of the top six internodes. Branch angle was defined as the angle at which the branch is inserted with respect to the primary stem at a particular node. A lateral appendage with at least one petiole was considered a branch. Branches were trimmed to approximately 30 mm from the primary stem and imaged with a blue background to capture the branch junction (Fig S7). While imaging, the blue background was held perpendicular to the camera to reduce parallax error. The angle at this junction was estimated using ImageJ. Mean branch angle per plant was calculated as well as mean branch angle per plot.

### Imaging and analysis of plant shape

To parameterize the outline shape in a height-normalized coordinate system, we fit an outline shape model to the measurements obtained from images of intact individual plants based on the probability density function of the beta-distribution to the values of *x* and *y* for each accession. With inclusion of an amplitude parameter (Sh_W) and re-parameterization, plant shape was described by three readily interpretable parameters: the maximum distance from the center line (Sh_W), the height for the maximum distance(Sh_H), and the area under the curve (Sh_A) (Note Methods S1 for further details of the shape model). Together with the plant height value, these four parameters describe the modeled outline shape of an individual plant of each accession.

### Light interception and CO_2_ assimilation

Light interception in the canopy of each field plot was estimated at noon using LI-191R Line Quantum Sensor (LI-COR Biosciences, NE, USA) at 100 mm increments from ground up to the top of the canopy using a previously described method [37]. Percent transmittance (tPAR) was calculated at each transect of the canopy as a ratio of transmitted PAR (TPAR) and incident PAR (iPAR) which is the radiation above the canopy [55, 56]. A logistical model was determined to be the best fit (based on adjusted R-squared values, p-values and lowest Akaike information criterion. Since the accessions in the study have different heights, we normalized LI to the plant height of 1 m. We plotted the relative light intensity (tPAR) and absorbance against normalized height, fit a logistic regression while fixing absorbance = 0 at height = 1 m. From the model, height of the plant at which 50% tPAR is reached and tPAR at 50% height of the plant was estimated. We also estimated CO_2_ assimilation at different heights along the canopy of different accessions by using the light response curve based on [38]. We used the LI data (PAR) to estimate the CO_2_ assimilation rate per plant row of a meter at different row heights for each accession (Note Methods S1 for further details).

### Canopy coverage

An unmanned aircraft system (UAS), DJI Inpsire 1, was used in this study to quantify CC [21]. From the logistic fit, we estimated accumulated/average canopy coverage ACC [30]. The fit was used to calculate rate of change in CC over a day (MCC_d) as well as a week (MCC_w) in a sliding window of seven days over the growing season. CC_50 was calculated as days to 50% canopy coverage for each accession from the fit.

### Data analysis

All data were analyzed using R (v4.0.2; R Core Team 2021) (Team, 2012). Summary statistics for all traits including mean, median, and standard deviation were calculated using the describeBy function within the psych package [57]. Best linear unbiased predictions (BLUPs) were calculated using the lmer function within the lme4 package [58]. A linear model was fit to the data that included the effects of year, block nested within year, accession (i.e., genotype), and accession-by-environment interaction. All effects were fit as random effects and residuals were assumed to be independent and identically distributed. Reliability (${i}_{ACC}^{2}$) of accession BLUPs for each trait in each year was estimated as ${i}_{ACC}^{2}={\widehat{\sigma }}_{G}^{2}/{(\widehat{\sigma }}_{G}^{2}+\frac{{\widehat{\sigma }}_{\epsilon }^{2}}{r)})$, where ${\widehat{\sigma }}_{G}^{2}$ is an estimate of the variance among accessions (the genotypic variance), ${\widehat{\sigma }}_{\epsilon }^{2}$ is an estimate of the residual variance, and *r* is the number of replicates, being four in 2018 and two in 2019 [59].

Pearson and Spearman correlation coefficients among all traits were calculated using the rcorr function within the Hmisc package (Table S4) [60]. A heatmap colored according to the Pearson correlation was created with the heatmap.2 function within the ggplots package [61].

The canopy cover heatmap (Fig. 6) was created using the heatmap.2 function using Z-scores calculated by applying the scale function to shoot architecture trait BLUPs to normalize BLUPs in relation to each other for visualization. Only shoot architecture traits that were significantly correlated with the canopy cover trait ACC (FDR-adjusted p-value < 0.05) were included in the heatmap. Accessions were ordered vertically in the heatmap by CC and horizontally by the strength of their correlation with ACC.

### Electronic supplementary material

Below is the link to the electronic supplementary material.

**Supplementary Material 1: Fig S1:** Canopy coverage of all the accessions in the study over the planting season in 2018 and 2019 modeled by logistic regression. All the accessions included in the study are represented as individual logistic regression lines. **Fig S2:** Variation between different genotypes in all traits measured in the study in years 2018 and 2019. Averages for each trait was calculated at plot level in 2018 and 2019 and plotted as bar graph. Traits shown are canopy coverage (CC) traits: average canopy coverage (ACC), days to 50% canopy coverage (CC50), canopy coverage at R2 (CCR2), max growth rate (%/week) (MCC_w) and max growth rate (%/day) (MCC_d); light interception (LI) traits: photosynthetically active radiation at 50 % plant height (PAR50H); plant height at 50% photosynthetically active radiation (H50PAR) and photosynthetically active radiation at Ground (PARG); plant shape parameters: maximum height normalized (Sh_H) , maximum width relative to height (Sh_W) and area under the curve (Sh_A); shoot architecture traits: node number (NO), branch number (BN), branching zone (BZ), branching ratio (BR), branch angle (BA), branching density (BD), branching orientation (BO), leaf length (LL), leaf width (LW), leaf area (LA), petiole length at node 4 (PL4), petiole slope at node 4 (PS4), petiole angle node 4 (PA4), internode length at node 4 (IL4) and internode slope at node 4 (IS4). Error bars are standard deviations from mean. **Fig S3:** Logistic function was used to model the light interception in different accessions: (a) The PAR values shown as relative light intensity measured along every 10 cm increment from bottom to top the plants in a row (Y axis) was plotted against the height of plant, normalized to 1 m height to account for variation in height between accessions (X axis). Logistic fit for each rep (different colored lines) as well as the mean fit (black solid line) are shown for each accession. 95% confidence interval for the fit is indicated (black dashed line). (b) The PAR data was converted to absorbance and plotted against normalized height for each accession. A logistic function was fit to the data. The mean values for the fit for each accession is shown as black solid line with the black dotted lines showing the 95% confidence intervals for the fit. **Fig S4:** Beta distribution function was used to express the shape of soybean plants: Width of the plant along the length of the plant was measured at 0, 12.5 25 37.5, 50, 65.5, 75, 87.5 and 100 % height of the plant. A beta distribution function was used to fit the width data to approximate the shape of the plant. Outcome of a beta distribution fit for each accession in the study is shown. The mean values for the fit for each plant in an accession is shown as black solid line with the black dotted lines showing the 95% confidence intervals for the fit. From the fit, the overall shape could be described in terms of three parameters: peak height (Sh_H), area under the curve (Sh_A), width scaling factor (Sh_W). **Fig S5:** An illustration depicting the assumptions made to simplify the CO2 assimilation rate estimation. (i) Plant row is approximated by a prism-like shape with the cross section outline of the outline shape model based on beta distribution with the three parameters peak height (Sh_H), area under the curve (Sh_A) and width scaling factor (Sh_W); (ii) the top-facing surface of the prism represents the photosynthetically active tissue shown in green (iii) the light comes from strait up shown as yellow arrows. **Fig S6:** Length of internodes and petioles from the top 4 nodes on the main stem of select accessions: a) Length of internodes were measured from the top four nodes of the accessions 5R22C11D, 5R14C48D, Bert and PI612732 and plotted on the y-axis. A linear regression was used to model the internode lengths for each accession and a slope was fitted based on the regression. The slopes for each accession are shown as a bar graph. Upper panel shows results from 2018 and the lower panel from 2019. On the right, pictures indicate the shape of two selected accessions showing most differences in their internode slope (IS4) values displaying variation for the shape at the top of the plant (indicated as yellow curves). b) Length of petioles were measured from the top four nodes of the accessions 5R22C11D, 5R14C48D, Bert and PI612732 and plotted on the y-axis. A linear regression was used to model the petiole lengths for each accession and a slope was fitted based on the regression. The slopes for each accession are shown as a bar graph. The upper panel shows results from 2018 and the lower panel from 2019. Pictures on the right show the petiole and internode images from the top of the plant. The petiole slope was derived from the top four petioles (PS4) and is indicated with a yellow arrow. **Fig S7:** Imaging for branch angle measurements. Junction of every primary branch on the main stem was imaged using a blue strip background to show the angle of branching. Each image was captured with the camera placed at a 90 degree angle to the blue background to avoid parallax error. Image J was used to measure the angle and average branch angle per plant was calculated

**Supplementary Material 2: Table S1:** List of Accessions used: FN designation indicates plants derived from a fast neutron mutagenized population

**Supplementary Material 3: Table S2:** Canopy coverage data from 2018 and 2019. Canopy coverage is expressed using a UAV and expressed on a scale of 0 to 1 indicating no coverage (0) to 100% coverage. Parameters calculated from the data are CC50 - fit time (days) reached half max canopy coverage (inflection point / max growth rate), MCC_d - interpolated max growth rate (%/day), MCC_w -(sliding window of 7 days) max rate (%%/week) from interpolated fit and ACC - average canopy accumulation [30]

**Supplementary Material 4: Table S3:** Reliability estimates, BLUPs calculated for the traits measured in this study and data associated with each trait measured in the study. Reliability and BLUPs were calculated for data obtained in 2018 and 2019. The traits measured are : Plant shape parameters area under the curve (Sh_A), Slope of the top four internodes (IS5), Plant shape parameter width scaling factor (Sh_W), Internode length at node 5 from the top of the plant (IL5), Internode length at node 4 from the top of the plant (IL4), max growth rate(%/week) (MCC_w), Canopy Height (CH), max growth rate (%/day) (MCC_d), Number of nodes (NO), Petiole angle at node 5 from the top of the plant (PA5), Leaf area (LA), Branch ratio (BR), Leaf Length (LL), Branch orientation (BO), Petiole angle at node 4 from the top of the plant (PA4), Plant shape parameter peak height (Sh_H), tPAR at 50% height (PAR50H), Height at 50% tPAR (H50PAR), tPAR at ground level (PARG), Branching zone (BZ), Branch density/ distribution (BD), Petiole length at node 4 from the top of the plant (PL4), Branch number (BN), Slope of the top four petioles (PS4), Leaf Width (LW), Petiole length at node 5 from the top of the plant (PL5), Days to 50% canopy coverage (CC50), Canopy coverage at R2 (CCR2), Average Canopy Coverage (ACC) and Branch angle (BA). Averages for all the traits measured in the study for 2018 and 2019 are shown

**Supplementary Material 5: Table S4:** Pearson?s and Spearman correlation coefficients and summary statistics for shoot architecture traits, canopy coverage and light interception traits measured in 2018 and 2019

**Supplementary Material 6: Table S5:** List of traits measured in this study and their definitions and abbreviations

**Supplementary Material 7: Table S6:** Weather data: Temperature and precipitation data from the 2001-2019 from St.Paul MN shown

Supplementary Material 8

## Data Availability

The data that supports the findings of this study are available in the Supporting Information of this article. The seeds for the fast neutron accessions mentioned in this article can be obtained by email requests to Bob Stupar at stup0004@umn.edu. The accessions can be requested from USDA Germplasm Resources Information Network (https://npgsweb.ars-grin.gov/gringlobal/search).
