# Supplementary material for: Variation in shoot architecture traits and their relationship to canopy coverage and light interception in soybean (Glycine max)
Source: BMC Plant Biol. 2024 Mar 16;24:194. doi: 10.1186/s12870-024-04859-2 (PMC10944616; doi:10.1186/s12870-024-04859-2)

Supporting information

Fig S1: Canopy coverage of all the lines in the study over the planting season in 2018 and 2019 modeled by logistic regression

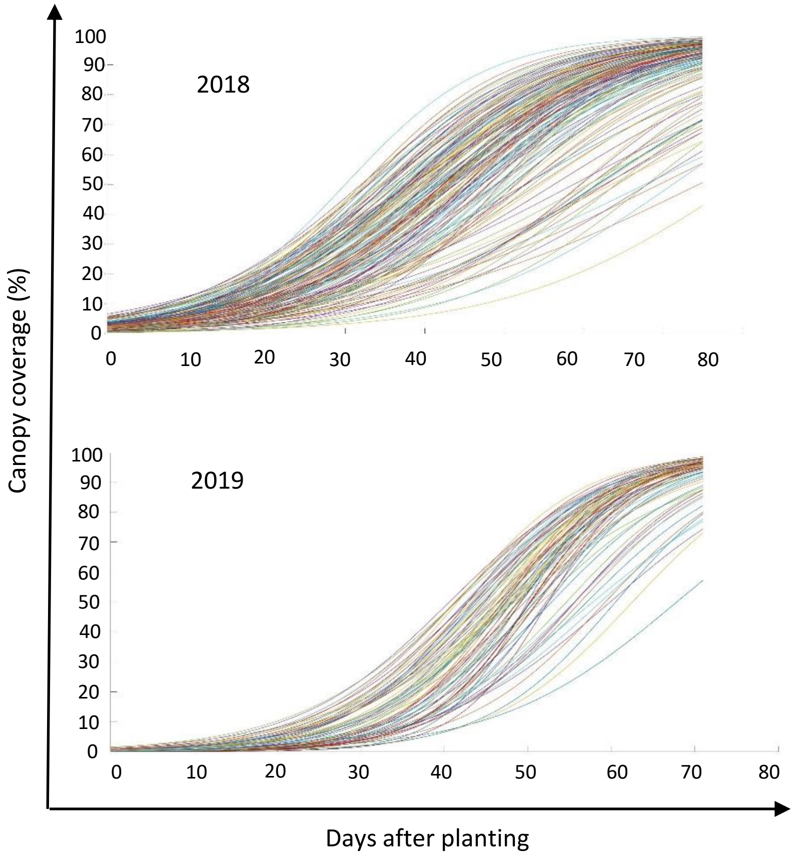

Fig S2 Variation between different genotypes in all traits measured in the study in years 2018 and 2019

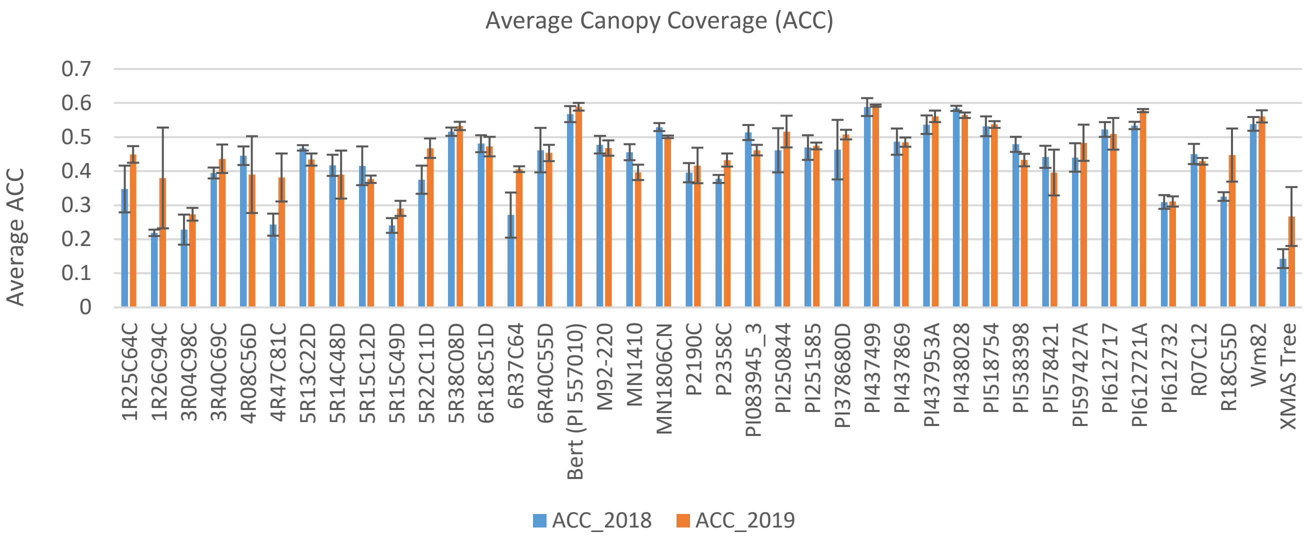

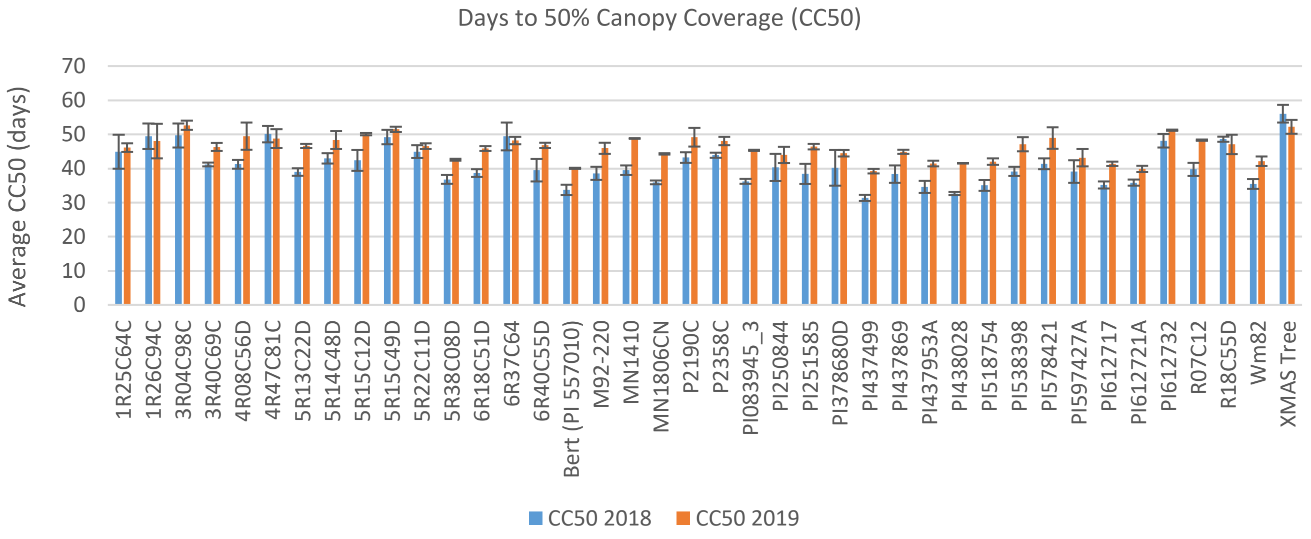

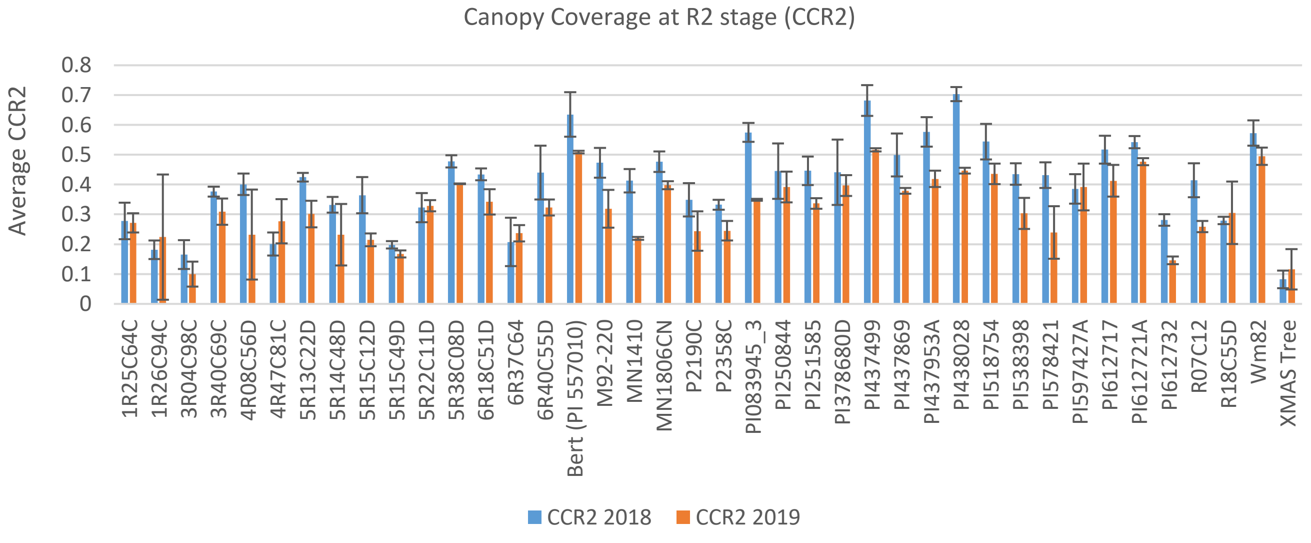

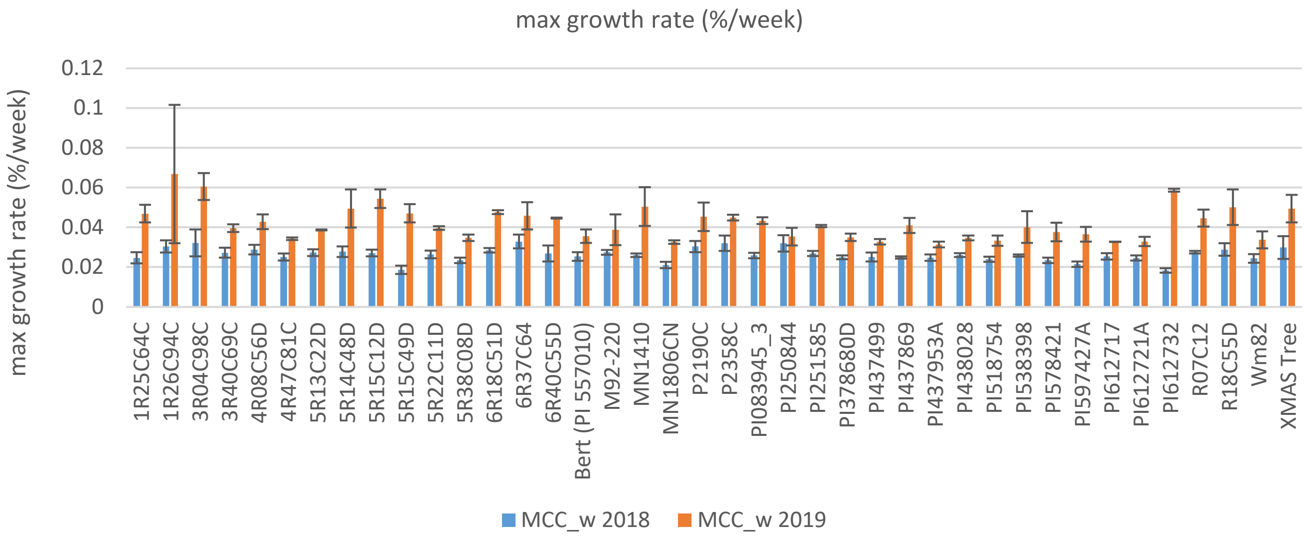
max growth rate (%/week)
max growth rate (%/week)
0.12
0.1
0.08
0.06
0.04
0.02
0
1R25C64C
1R26C94C
3R04C98C
3R40C69C
4R08C56D
4R47C81C
5R13C22D
5R14C48D
5R15C12D
5R15C49D
5R22C11D
5R38C08D
6R18C51D
6R37C64
6R40C55D
Bert (PI 557010)
M92-220
MN1410
MN1806CN
P2190C
P2358C
PI083945_3
PI250844
PI251585
PI378680D
PI437499
PI437869
PI437953A
PI438028
PI518754
PI538398
PI578421
PI597427A
PI612717
PI612721A
PI612732
R07C12
R18C55D
Wm82
XMAS Tree
MCC_w 2018
MCC_w 2019

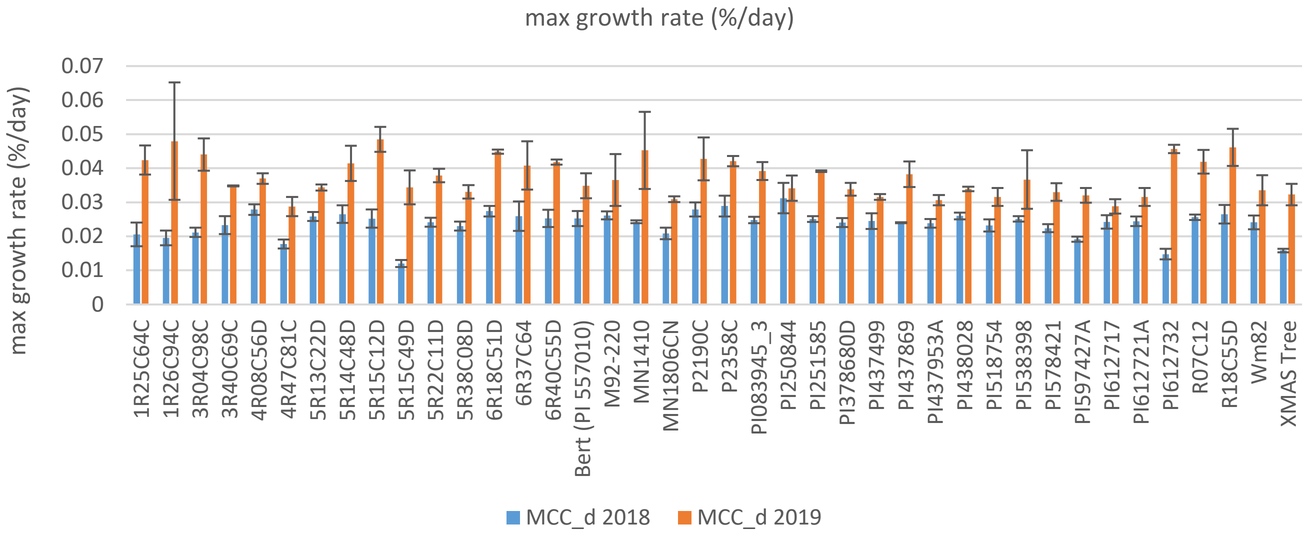
max growth rate (%/day)
max growth rate (%/day)
0.07
0.06
0.05
0.04
0.03
0.02
0.01
0
1R25C64C
1R26C94C
3R04C98C
3R40C69C
4R08C56D
4R47C81C
5R13C22D
5R14C48D
5R15C12D
5R15C49D
5R22C11D
5R38C08D
6R18C51D
6R37C64
6R40C55D
Bert (PI 557010)
M92-220
MN1410
MN1806CN
P2190C
P2358C
PI083945_3
PI250844
PI251585
PI378680D
PI437499
PI437869
PI437953A
PI438028
PI518754
PI538398
PI578421
PI597427A
PI612717
PI612721A
PI612732
R07C12
R18C55D
Wm82
XMAS Tree
MCC_d 2018
MCC_d 2019

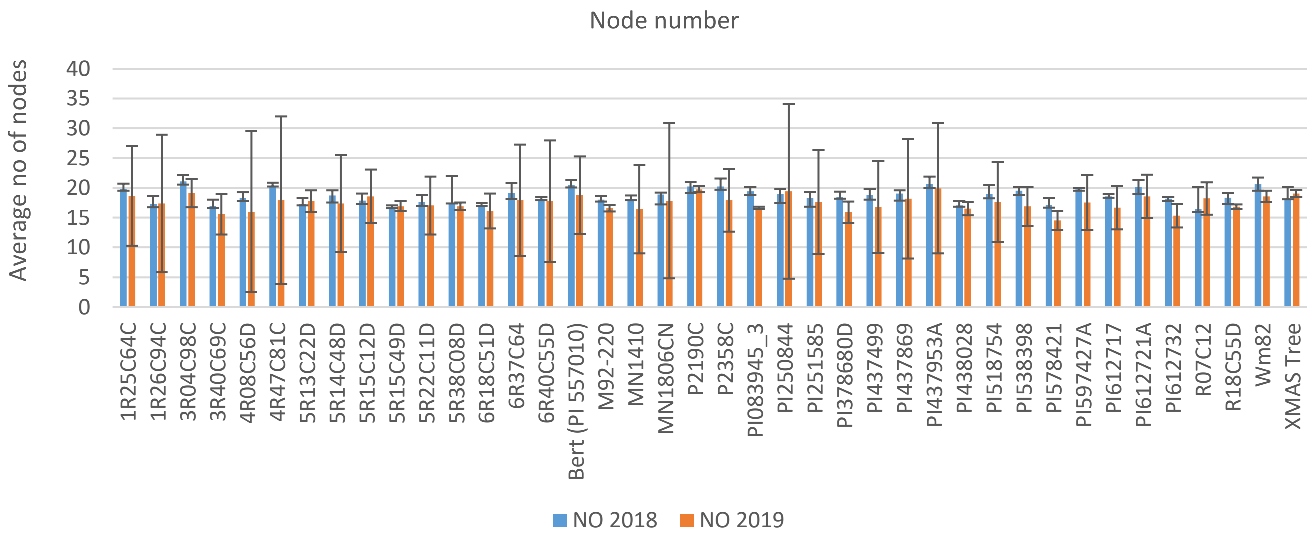
Node number
Average no of nodes
40
35
30
25
20
15
10
5
0
1R25C64C
1R26C94C
3R04C98C
3R40C69C
4R08C56D
4R47C81C
5R13C22D
5R14C48D
5R15C12D
5R15C49D
5R22C11D
5R38C08D
6R18C51D
6R37C64
6R40C55D
Bert (PI 557010)
M92-220
MN1410
MN1806CN
P2190C
P2358C
PI083945_3
PI250844
PI251585
PI378680D
PI437499
PI437869
PI437953A
PI438028
PI518754
PI538398
PI578421
PI597427A
PI612717
PI612721A
PI612732
R07C12
R18C55D
Wm82
XMAS Tree
NO 2018
NO 2019

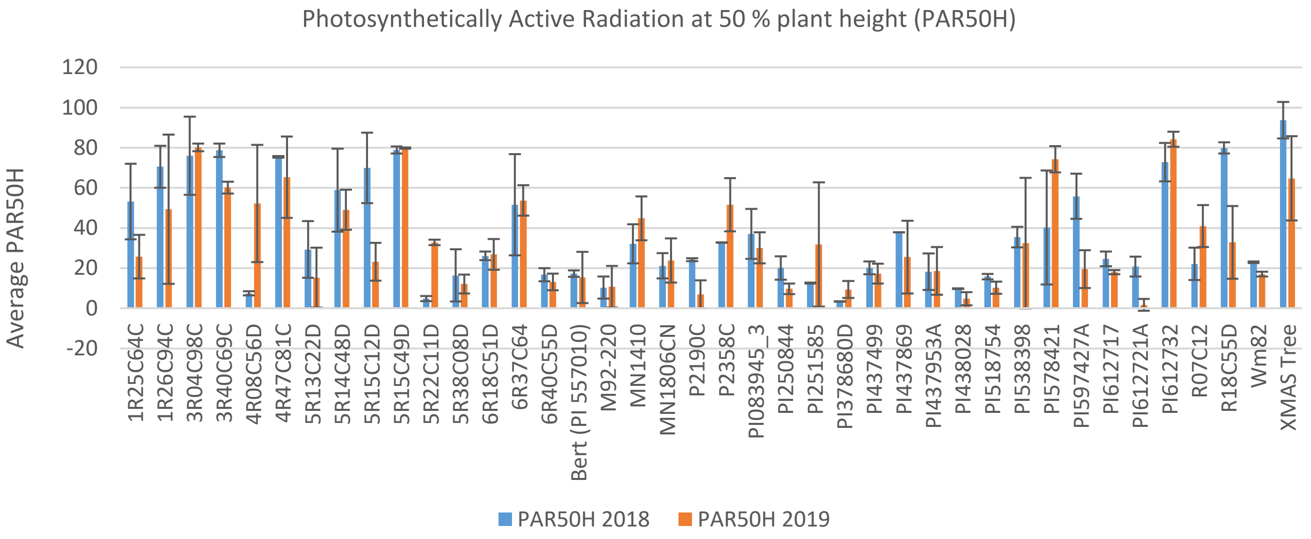
Photosynthetically Active Radiation at 50 % plant height (PAR50H)
Average PAR50H
120
100
80
60
40
20
0
-20
1R25C64C
1R26C94C
3R04C98C
3R40C69C
4R08C56D
4R47C81C
5R13C22D
5R14C48D
5R15C12D
5R15C49D
5R22C11D
5R38C08D
6R18C51D
6R37C64
6R40C55D
Bert (PI 557010)
M92-220
MN1410
MN1806CN
P2190C
P2358C
PI083945_3
PI250844
PI251585
PI378680D
PI437499
PI437869
PI437953A
PI438028
PI518754
PI538398
PI578421
PI597427A
PI612717
PI612721A
PI612732
R07C12
R18C55D
Wm82
XMAS Tree
PAR50H 2018
PAR50H 2019

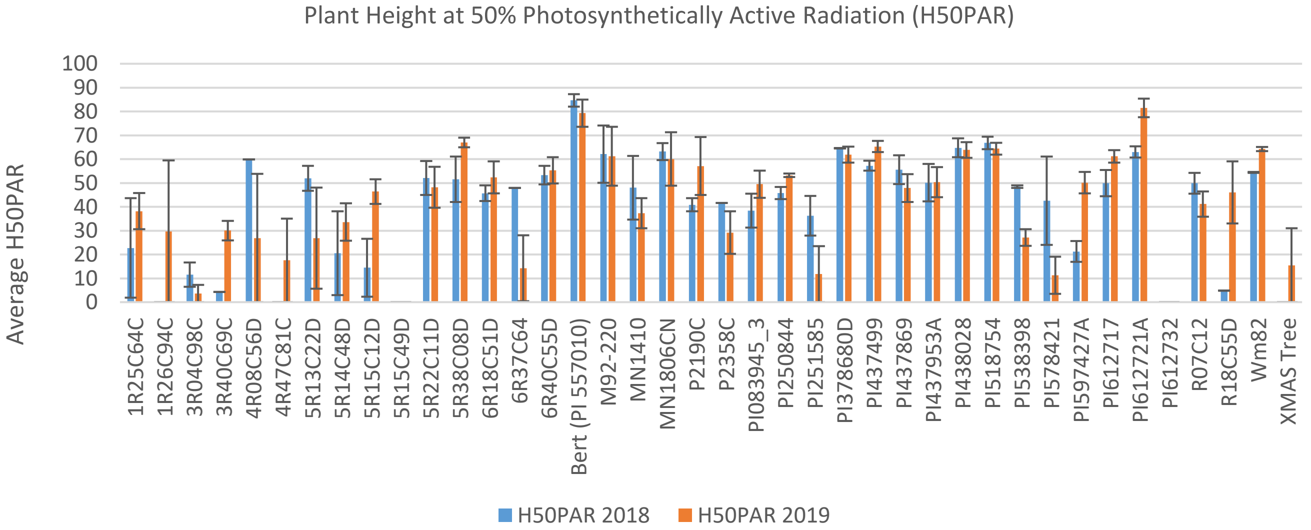
Plant Height at 50% Photosynthetically Active Radiation (H50PAR)
Average H50PAR
100
90
80
70
60
50
40
30
20
10
0
1R25C64C
1R26C94C
3R04C98C
3R40C69C
4R08C56D
4R47C81C
5R13C22D
5R14C48D
5R15C12D
5R15C49D
5R22C11D
5R38C08D
6R18C51D
6R37C64
6R40C55D
Bert (PI 557010)
M92-220
MN1410
MN1806CN
P2190C
P2358C
PI083945_3
PI250844
PI251585
PI378680D
PI437499
PI437869
PI437953A
PI438028
PI518754
PI538398
PI578421
PI597427A
PI612717
PI612721A
PI612732
R07C12
R18C55D
Wm82
XMAS Tree
H50PAR 2018
H50PAR 2019

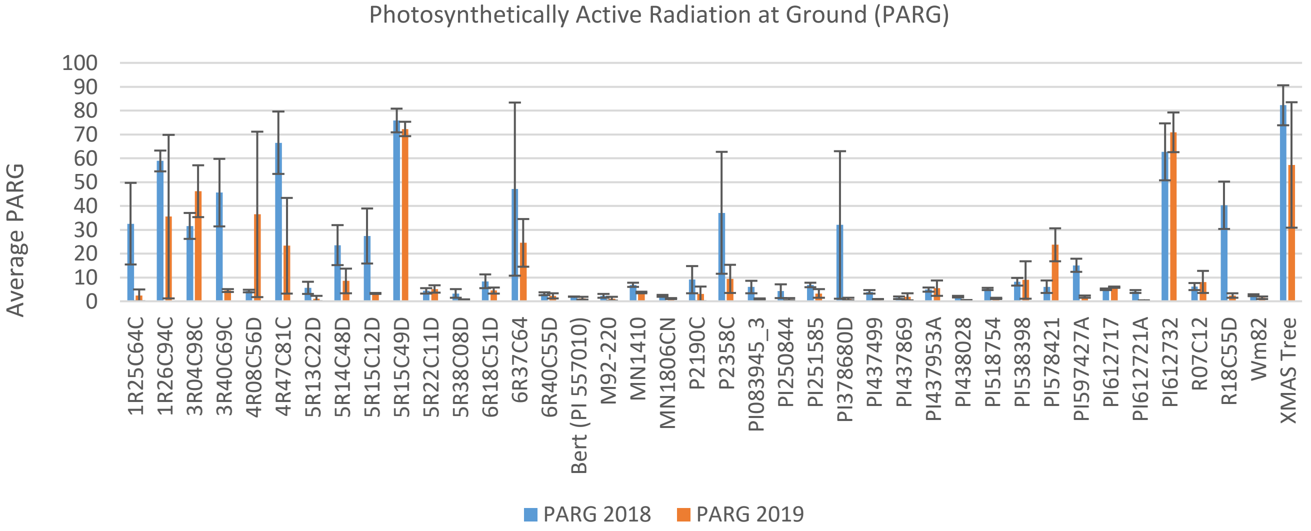
Photosynthetically Active Radiation at Ground (PARG)
Average PARG
100
90
80
70
60
50
40
30
20
10
0
1R25C64C
1R26C94C
3R04C98C
3R40C69C
4R08C56D
4R47C81C
5R13C22D
5R14C48D
5R15C12D
5R15C49D
5R22C11D
5R38C08D
6R18C51D
6R37C64
6R40C55D
Bert (PI 557010)
M92-220
MN1410
MN1806CN
P2190C
P2358C
PI083945_3
PI250844
PI251585
PI378680D
PI437499
PI437869
PI437953A
PI438028
PI518754
PI538398
PI578421
PI597427A
PI612717
PI612721A
PI612732
R07C12
R18C55D
Wm82
XMAS Tree
PARG 2018
PARG 2019

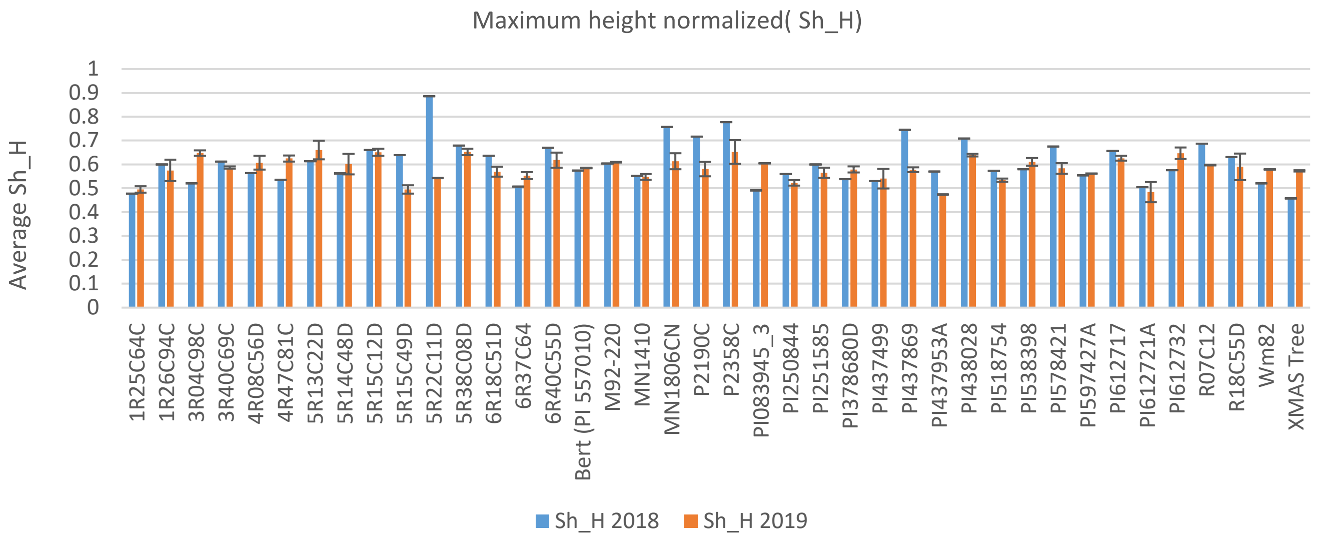
Maximum height normalized(Sh_H)
Average Sh_H
0
0.1
0.2
0.3
0.4
0.5
0.6
0.7
0.8
0.9
1
1R25C64C
1R26C94C
3R04C98C
3R40C69C
4R08C56D
4R47C81C
5R13C22D
5R14C48D
5R15C12D
5R15C49D
5R22C11D
5R38C08D
6R18C51D
6R37C64
6R40C55D
Bert (PI 557010)
M92-220
MN1410
MN1806CN
P2190C
P2358C
PI083945_3
PI250844
PI251585
PI378680D
PI437499
PI437869
PI437953A
PI438028
PI518754
PI538398
PI578421
PI597427A
PI612717
PI612721A
PI612732
R07C12
R18C55D
Wm82
XMAS Tree
Sh_H 2018
Sh_H 2019

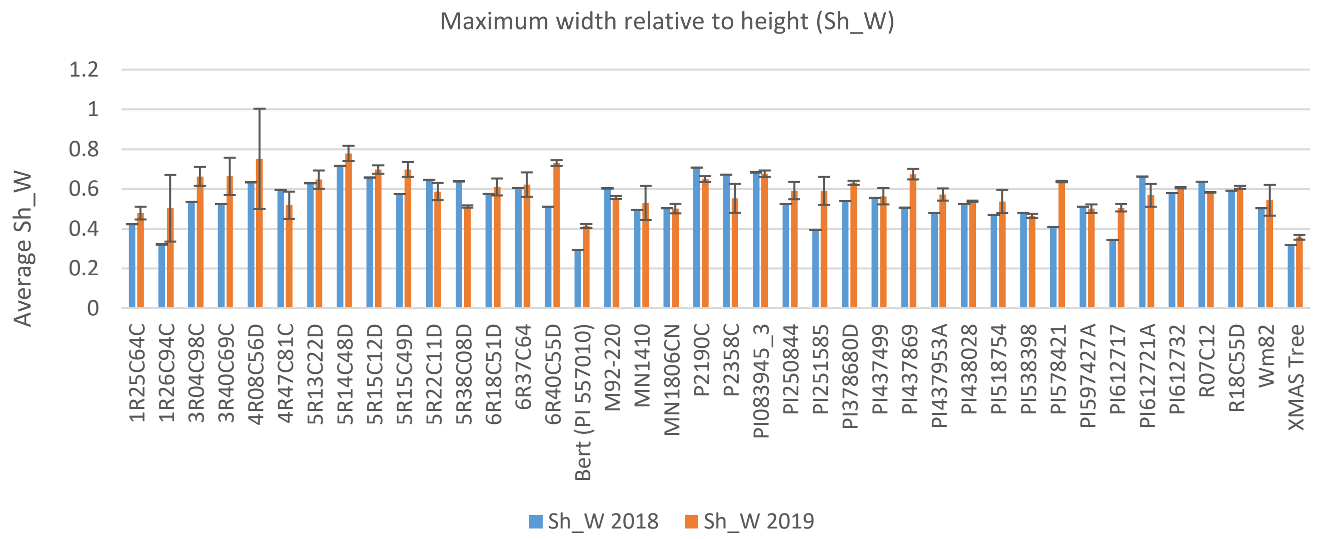
Maximum width relative to height (Sh_W)
Average Sh_W
0
0.2
0.4
0.6
0.8
1
1.2
1R25C64C
1R26C94C
3R04C98C
3R40C69C
4R08C56D
4R47C81C
5R13C22D
5R14C48D
5R15C12D
5R15C49D
5R22C11D
5R38C08D
6R18C51D
6R37C64
6R40C55D
Bert (PI 557010)
M92-220
MN1410
MN1806CN
P2190C
P2358C
PI083945_3
PI250844
PI251585
PI378680D
PI437499
PI437869
PI437953A
PI438028
PI518754
PI538398
PI578421
PI597427A
PI612717
PI612721A
PI612732
R07C12
R18C55D
Wm82
XMAS Tree
Sh_W 2018
Sh_W 2019

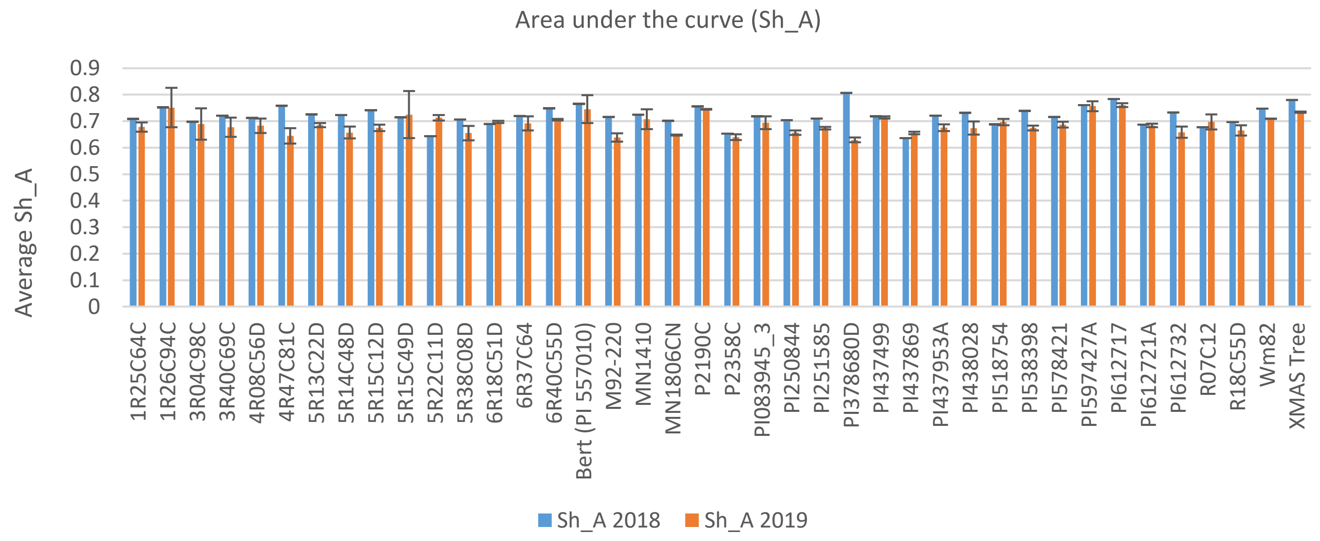
Area under the curve (Sh_A)
Average Sh_A
0
0.1
0.2
0.3
0.4
0.5
0.6
0.7
0.8
0.9
1R25C64C
1R26C94C
3R04C98C
3R40C69C
4R08C56D
4R47C81C
5R13C22D
5R14C48D
5R15C12D
5R15C49D
5R22C11D
5R38C08D
6R18C51D
6R37C64
6R40C55D
Bert (PI 557010)
M92-220
MN1410
MN1806CN
P2190C
P2358C
PI083945_3
PI250844
PI251585
PI378680D
PI437499
PI437869
PI437953A
PI438028
PI518754
PI538398
PI578421
PI597427A
PI612717
PI612721A
PI612732
R07C12
R18C55D
Wm82
XMAS Tree
Sh_A 2018
Sh_A 2019

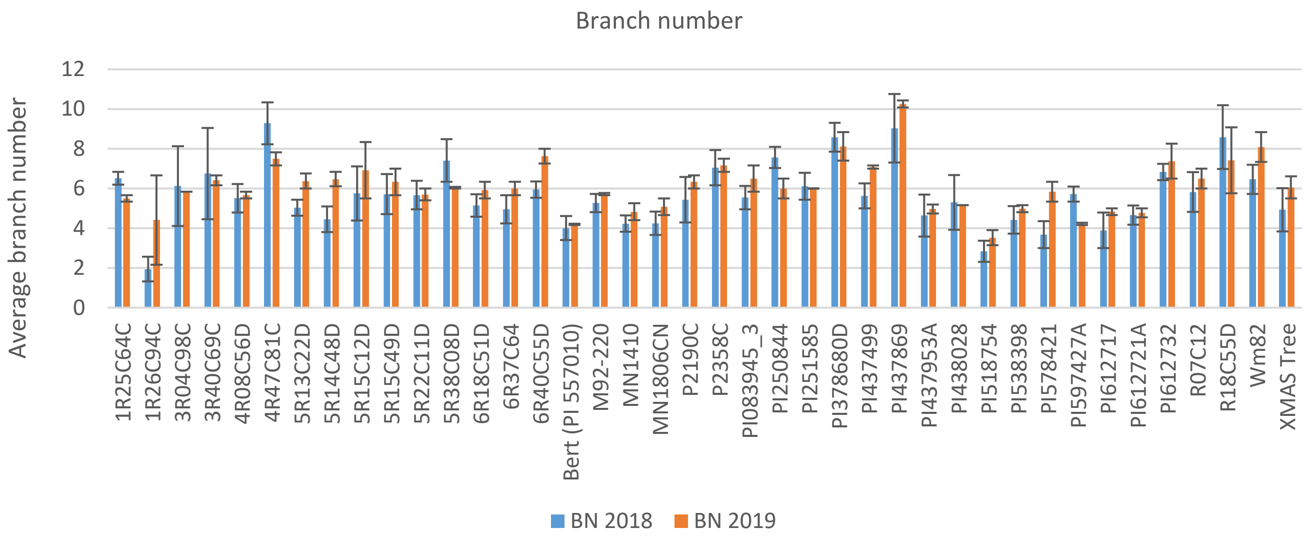
Branch number
Average branch number
BN 2018
BN 2019

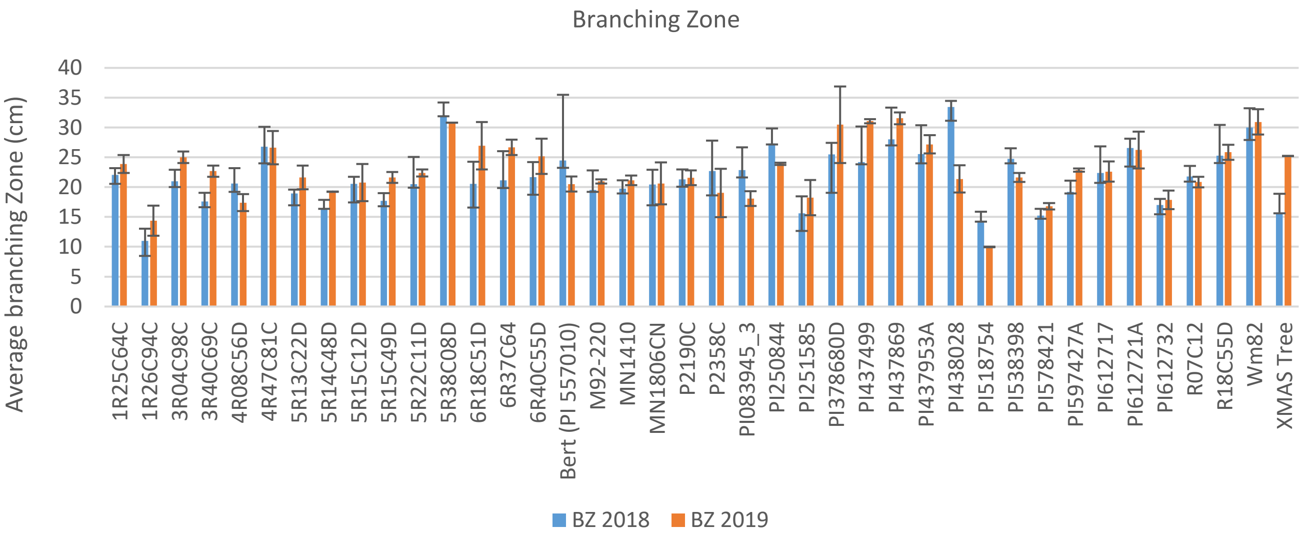
Branching Zone
Average branching Zone (cm)
BZ 2018
BZ 2019

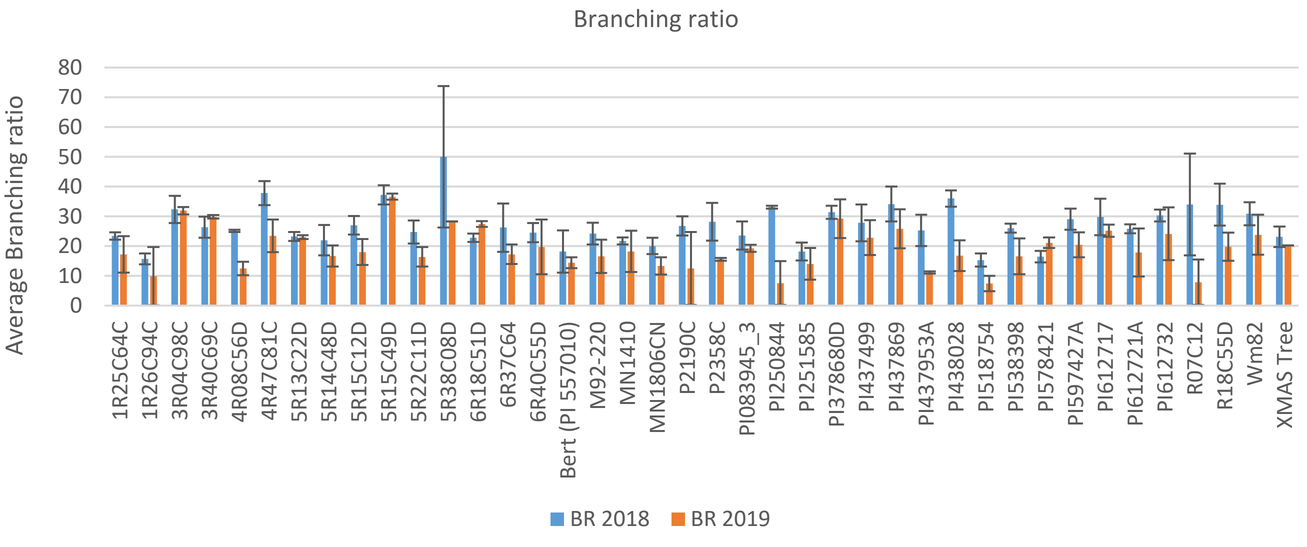
Branching ratio
Average Branching ratio
BR 2018
BR 2019

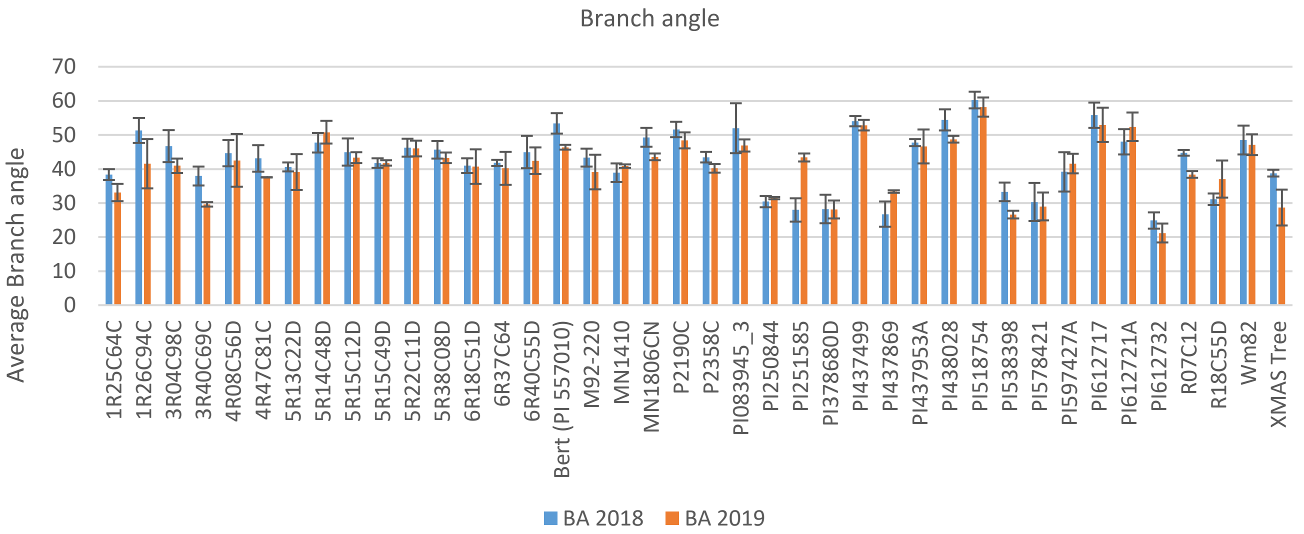
Branch angle
Average Branch angle
70
60
50
40
30
20
10
0
1R25C64C
1R26C94C
3R04C98C
3R40C69C
4R08C56D
4R47C81C
5R13C22D
5R14C48D
5R15C12D
5R15C49D
5R22C11D
5R38C08D
6R18C51D
6R37C64
6R40C55D
Bert (PI 557010)
M92-220
MN1410
MN1806CN
P2190C
P2358C
PI083945_3
PI250844
PI251585
PI378680D
PI437499
PI437869
PI437953A
PI438028
PI518754
PI538398
PI578421
PI597427A
PI612717
PI612721A
PI612732
R07C12
R18C55D
Wm82
XMAS Tree
BA 2018
BA 2019

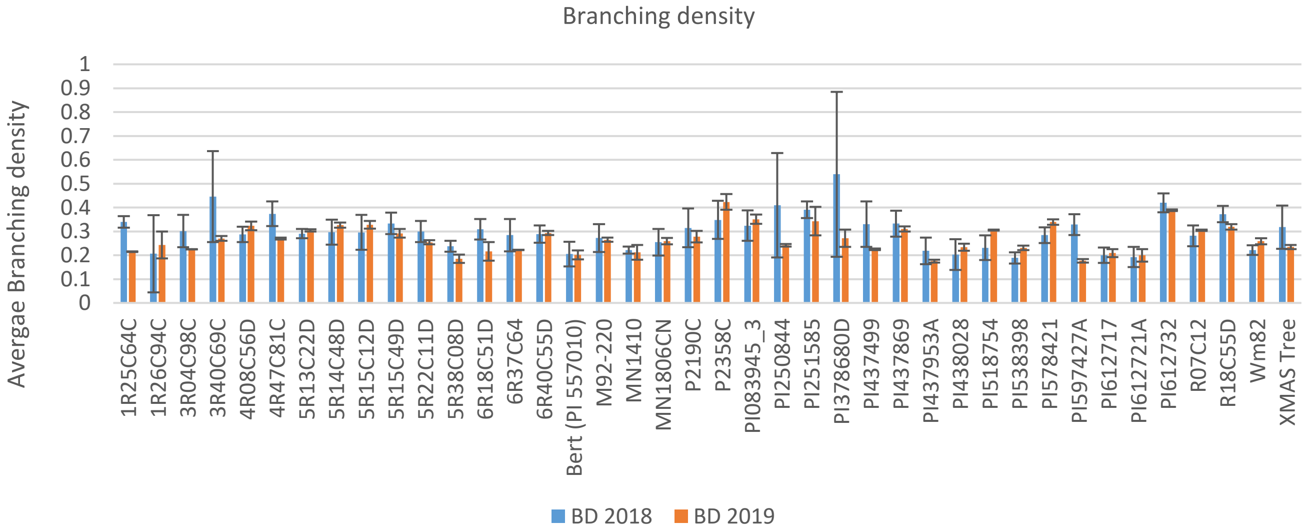
Branching density
Avergae Branching density
1
0.9
0.8
0.7
0.6
0.5
0.4
0.3
0.2
0.1
0
1R25C64C
1R26C94C
3R04C98C
3R40C69C
4R08C56D
4R47C81C
5R13C22D
5R14C48D
5R15C12D
5R15C49D
5R22C11D
5R38C08D
6R18C51D
6R37C64
6R40C55D
Bert (PI 557010)
M92-220
MN1410
MN1806CN
P2190C
P2358C
PI083945_3
PI250844
PI251585
PI378680D
PI437499
PI437869
PI437953A
PI438028
PI518754
PI538398
PI578421
PI597427A
PI612717
PI612721A
PI612732
R07C12
R18C55D
Wm82
XMAS Tree
BD 2018
BD 2019

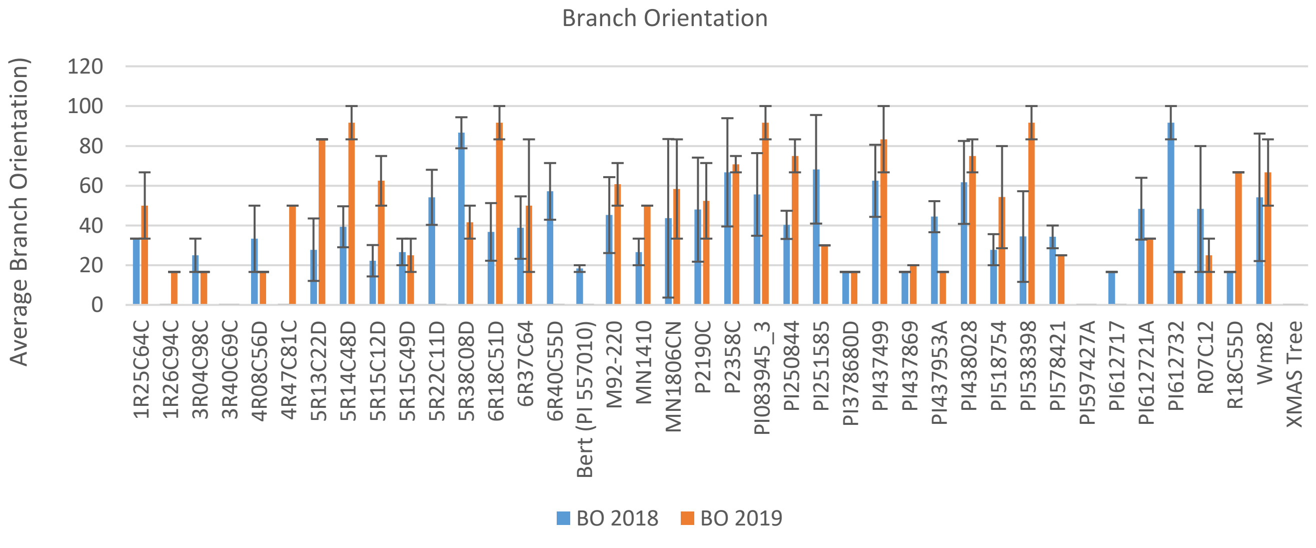
Branch Orientation
Average Branch Orientation)
120
100
80
60
40
20
0
1R25C64C
1R26C94C
3R04C98C
3R40C69C
4R08C56D
4R47C81C
5R13C22D
5R14C48D
5R15C12D
5R15C49D
5R22C11D
5R38C08D
6R18C51D
6R37C64
6R40C55D
Bert (PI 557010)
M92-220
MN1410
MN1806CN
P2190C
P2358C
PI083945_3
PI250844
PI251585
PI378680D
PI437499
PI437869
PI437953A
PI438028
PI518754
PI538398
PI578421
PI597427A
PI612717
PI612721A
PI612732
R07C12
R18C55D
Wm82
XMAS Tree
BO 2018
BO 2019

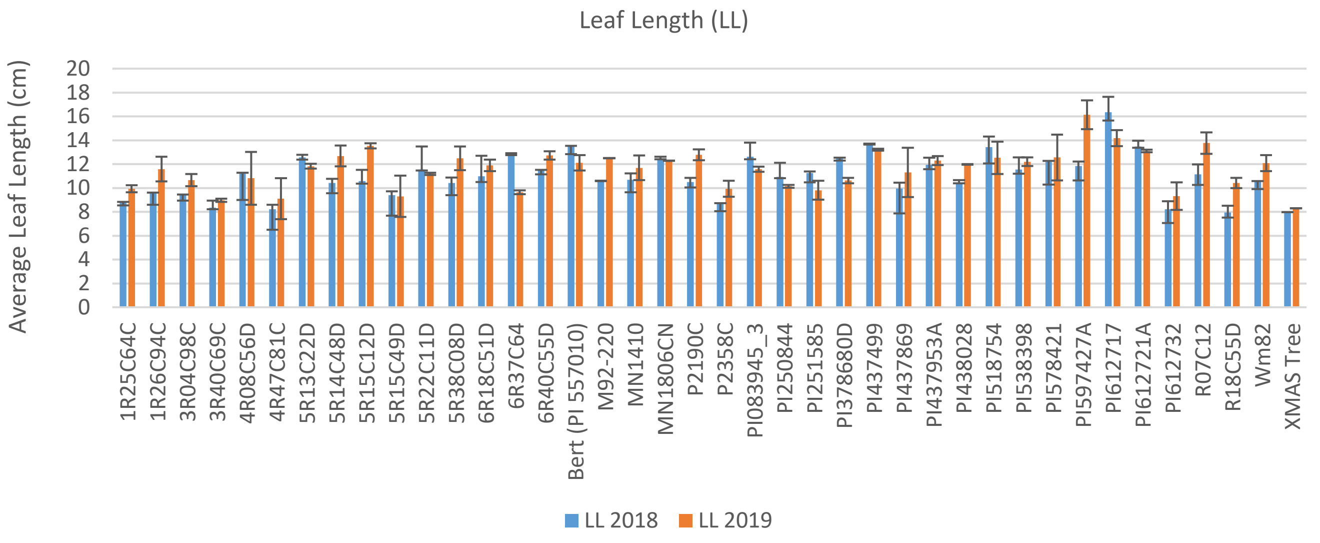
Leaf Length (LL)
Average Leaf Length (cm)
0
2
4
6
8
10
12
14
16
18
20
1R25C64C
1R26C94C
3R04C98C
3R40C69C
4R08C56D
4R47C81C
5R13C22D
5R14C48D
5R15C12D
5R15C49D
5R22C11D
5R38C08D
6R18C51D
6R37C64
6R40C55D
Bert (PI 557010)
M92-220
MN1410
MN1806CN
P2190C
P2358C
PI083945_3
PI250844
PI251585
PI378680D
PI437499
PI437869
PI437953A
PI438028
PI518754
PI538398
PI578421
PI597427A
PI612717
PI612721A
PI612732
R07C12
R18C55D
Wm82
XMAS Tree
LL 2018
LL 2019

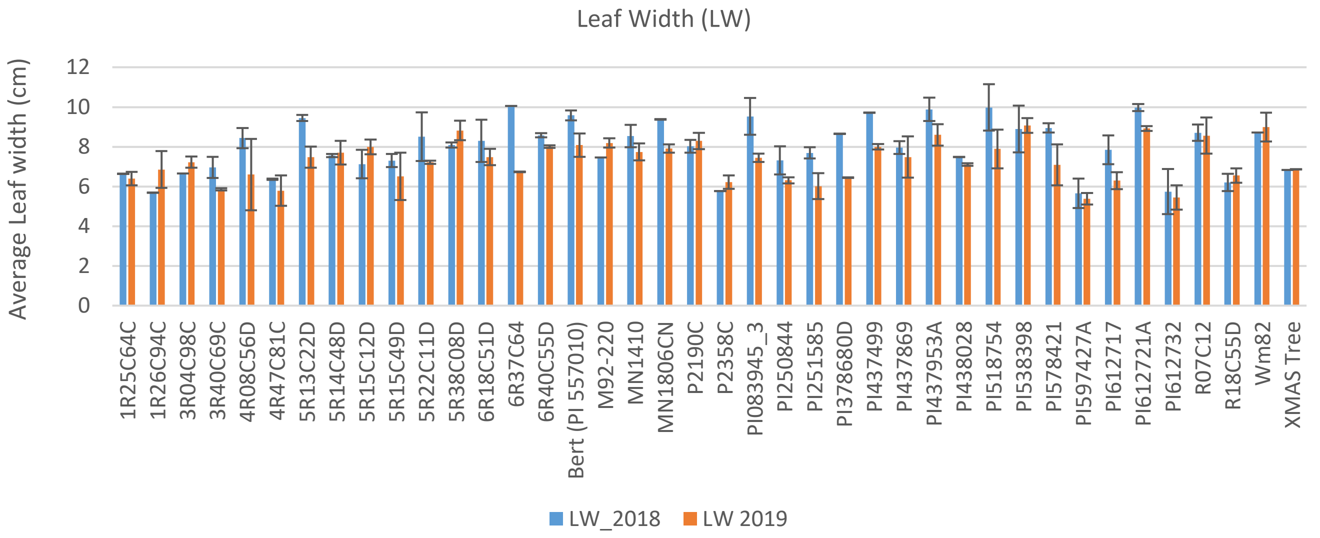
Leaf Width (LW)
Average Leaf width (cm)
0
2
4
6
8
10
12
1R25C64C
1R26C94C
3R04C98C
3R40C69C
4R08C56D
4R47C81C
5R13C22D
5R14C48D
5R15C12D
5R15C49D
5R22C11D
5R38C08D
6R18C51D
6R37C64
6R40C55D
Bert (PI 557010)
M92-220
MN1410
MN1806CN
P2190C
P2358C
PI083945_3
PI250844
PI251585
PI378680D
PI437499
PI437869
PI437953A
PI438028
PI518754
PI538398
PI578421
PI597427A
PI612717
PI612721A
PI612732
R07C12
R18C55D
Wm82
XMAS Tree
LW_2018
LW 2019

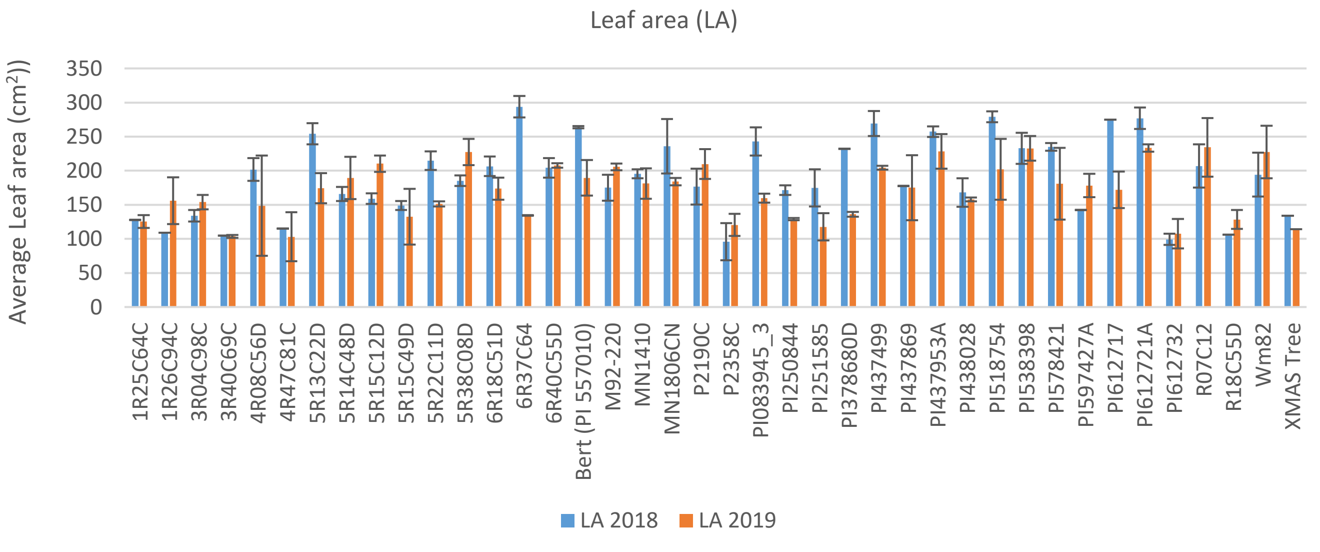
Leaf area (LA)
Average Leaf area (cm²))
0
50
100
150
200
250
300
350
1R25C64C
1R26C94C
3R04C98C
3R40C69C
4R08C56D
4R47C81C
5R13C22D
5R14C48D
5R15C12D
5R15C49D
5R22C11D
5R38C08D
6R18C51D
6R37C64
6R40C55D
Bert (PI 557010)
M92-220
MN1410
MN1806CN
P2190C
P2358C
PI083945_3
PI250844
PI251585
PI378680D
PI437499
PI437869
PI437953A
PI438028
PI518754
PI538398
PI578421
PI597427A
PI612717
PI612721A
PI612732
R07C12
R18C55D
Wm82
XMAS Tree
LA 2018
LA 2019

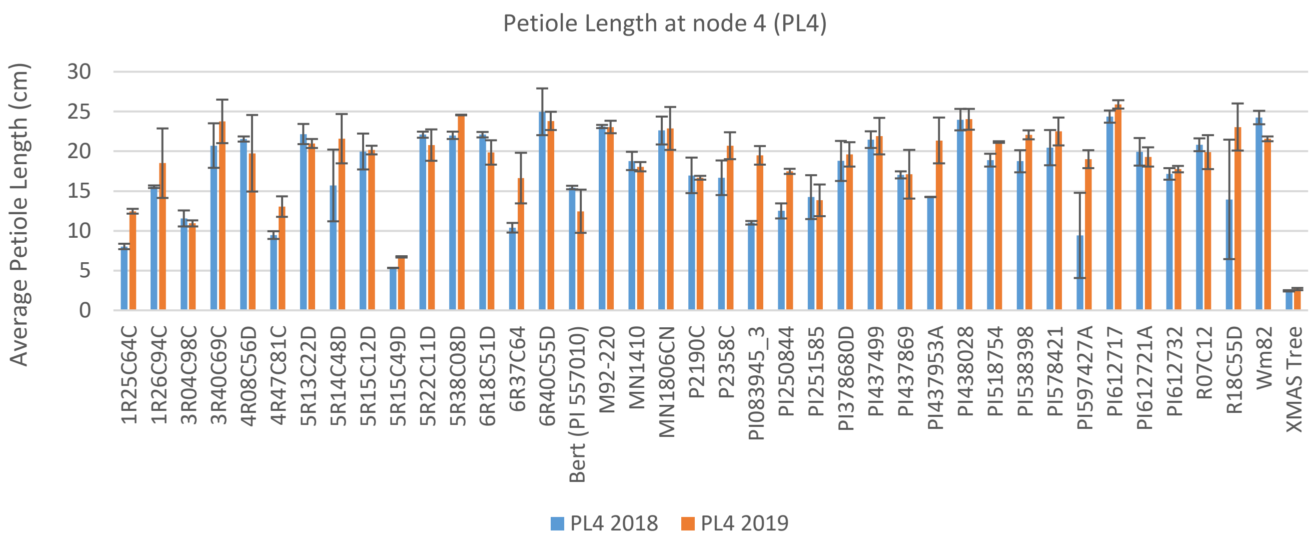
Petiole Length at node 4 (PL4)
Average Petiole Length (cm)
1R25C64C
1R26C94C
3R04C98C
3R40C69C
4R08C56D
4R47C81C
5R13C22D
5R14C48D
5R15C12D
5R15C49D
5R22C11D
5R38C08D
6R18C51D
6R37C64
6R40C55D
Bert (PI 557010)
M92-220
MN1410
MN1806CN
P2190C
P2358C
PI083945_3
PI250844
PI251585
PI378680D
PI437499
PI437869
PI437953A
PI438028
PI518754
PI538398
PI578421
PI597427A
PI612717
PI612721A
PI612732
R07C12
R18C55D
Wm82
XMAS Tree
PL4 2018
PL4 2019

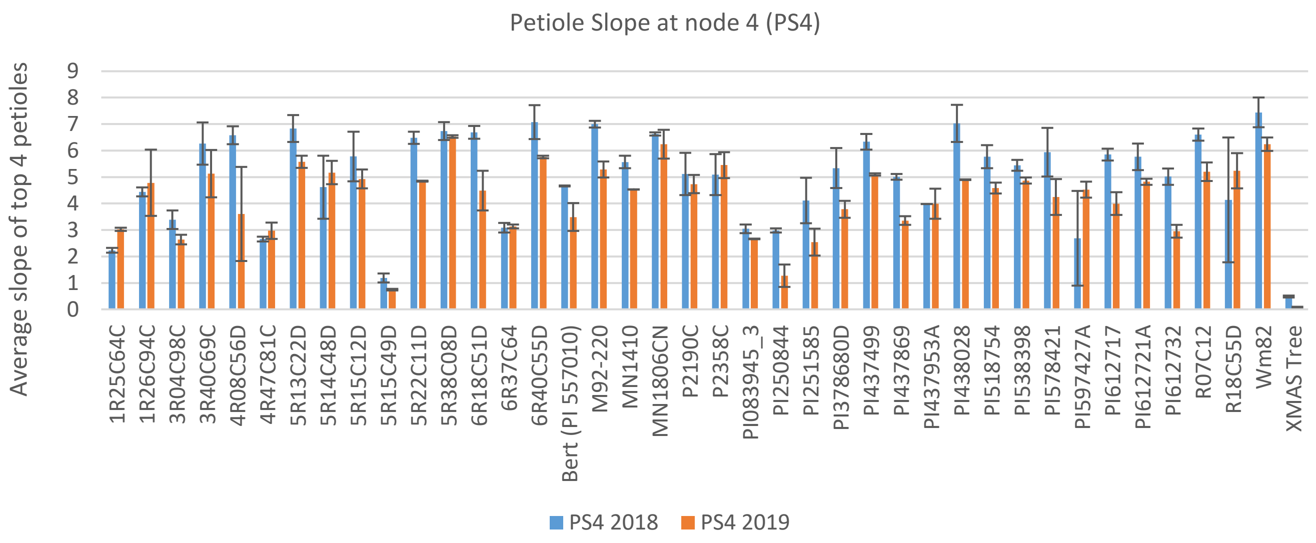
Petiole Slope at node 4 (PS4)
Average slope of top 4 petioles
1R25C64C
1R26C94C
3R04C98C
3R40C69C
4R08C56D
4R47C81C
5R13C22D
5R14C48D
5R15C12D
5R15C49D
5R22C11D
5R38C08D
6R18C51D
6R37C64
6R40C55D
Bert (PI 557010)
M92-220
MN1410
MN1806CN
P2190C
P2358C
PI083945_3
PI250844
PI251585
PI378680D
PI437499
PI437869
PI437953A
PI438028
PI518754
PI538398
PI578421
PI597427A
PI612717
PI612721A
PI612732
R07C12
R18C55D
Wm82
XMAS Tree
PS4 2018
PS4 2019

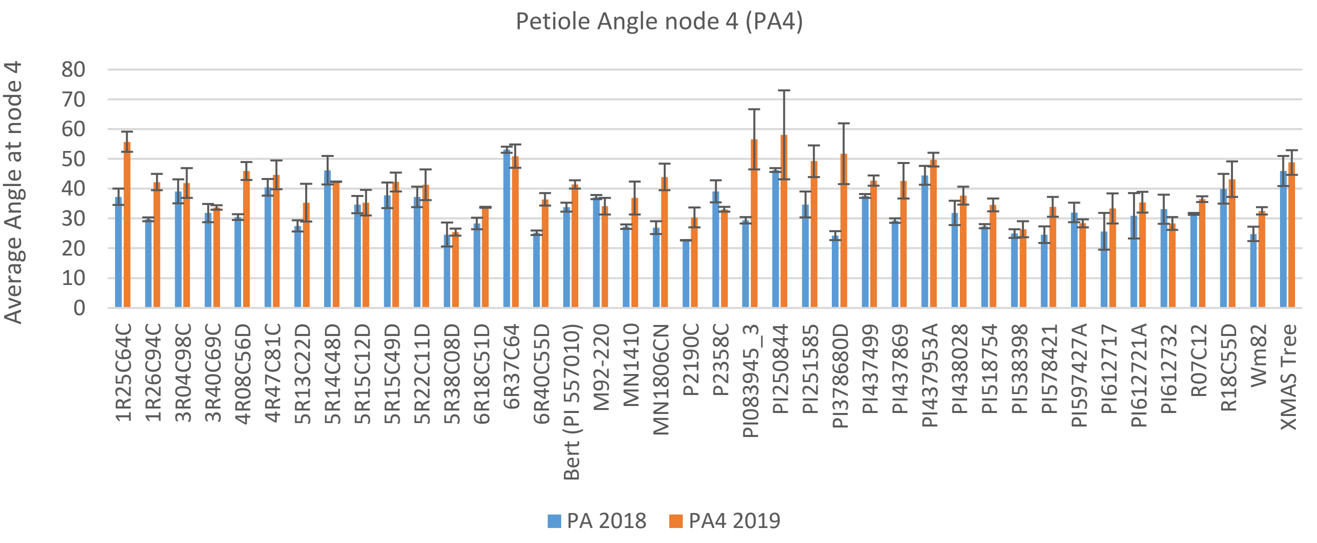
Petiole Angle node 4 (PA4)
Average Angle at node 4
1R25C64C
1R26C94C
3R04C98C
3R40C69C
4R08C56D
4R47C81C
5R13C22D
5R14C48D
5R15C12D
5R15C49D
5R22C11D
5R38C08D
6R18C51D
6R37C64
6R40C55D
Bert (PI 557010)
M92-220
MN1410
MN1806CN
P2190C
P2358C
PI083945_3
PI250844
PI251585
PI378680D
PI437499
PI437869
PI437953A
PI438028
PI518754
PI538398
PI578421
PI597427A
PI612717
PI612721A
PI612732
R07C12
R18C55D
Wm82
XMAS Tree
PA 2018
PA4 2019

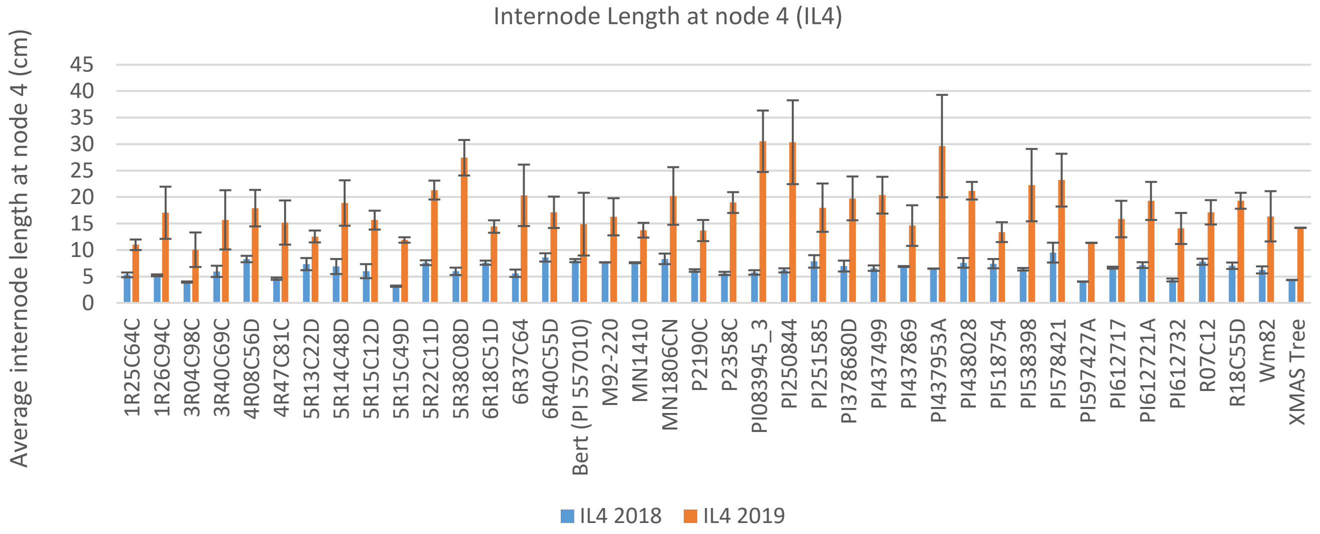
Internode Length at node 4 (IL4)
Average internode length at node 4 (cm)
45
40
35
30
25
20
15
10
5
0
1R25C64C
1R26C94C
3R04C98C
3R40C69C
4R08C56D
4R47C81C
5R13C22D
5R14C48D
5R15C12D
5R15C49D
5R22C11D
5R38C08D
6R18C51D
6R37C64
6R40C55D
Bert (PI 557010)
M92-220
MN1410
MN1806CN
P2190C
P2358C
PI083945_3
PI250844
PI251585
PI378680D
PI437499
PI437869
PI437953A
PI438028
PI518754
PI538398
PI578421
PI597427A
PI612717
PI612721A
PI612732
R07C12
R18C55D
Wm82
XMAS Tree
IL4 2018
IL4 2019

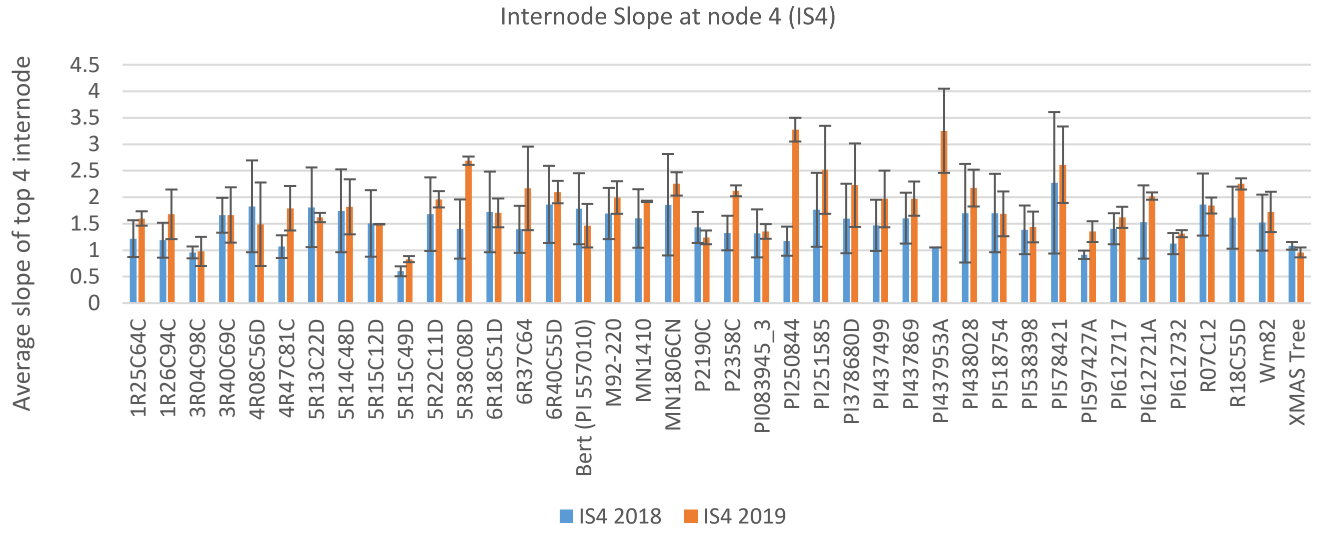
Internode Slope at node 4 (IS4)
Average slope of top 4 internode
4.5
4
3.5
3
2.5
2
1.5
1
0.5
0
1R25C64C
1R26C94C
3R04C98C
3R40C69C
4R08C56D
4R47C81C
5R13C22D
5R14C48D
5R15C12D
5R15C49D
5R22C11D
5R38C08D
6R18C51D
6R37C64
6R40C55D
Bert (PI 557010)
M92-220
MN1410
MN1806CN
P2190C
P2358C
PI083945_3
PI250844
PI251585
PI378680D
PI437499
PI437869
PI437953A
PI438028
PI518754
PI538398
PI578421
PI597427A
PI612717
PI612721A
PI612732
R07C12
R18C55D
Wm82
XMAS Tree
IS4 2018
IS4 2019

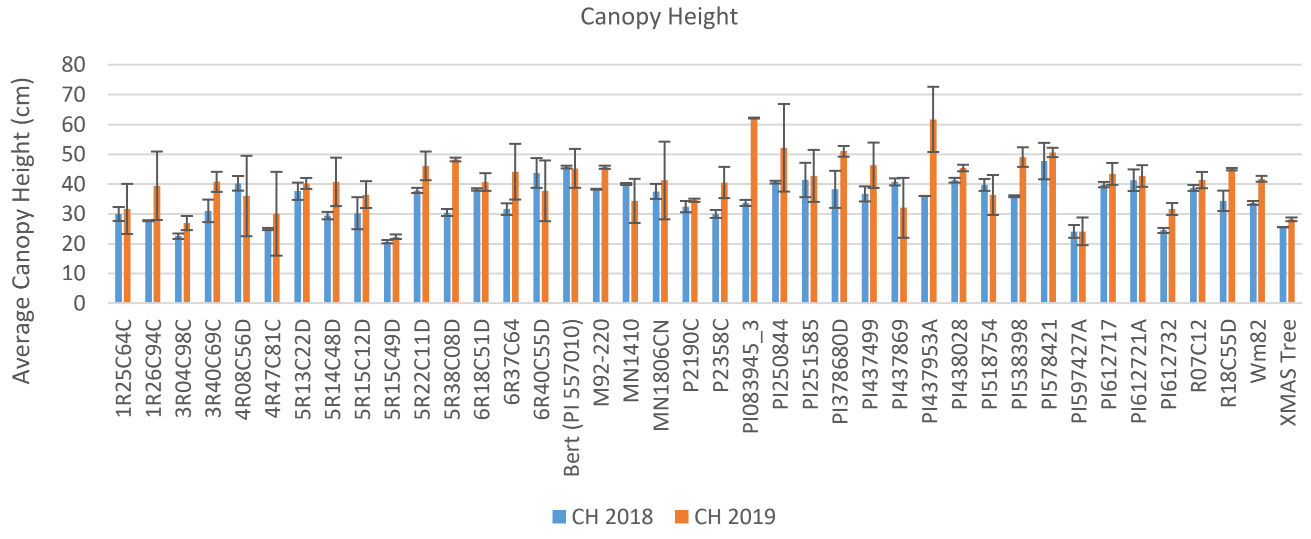
Canopy Height
Average Canopy Height (cm)
80
70
60
50
40
30
20
10
0
1R25C64C
1R26C94C
3R04C98C
3R40C69C
4R08C56D
4R47C81C
5R13C22D
5R14C48D
5R15C12D
5R15C49D
5R22C11D
5R38C08D
6R18C51D
6R37C64
6R40C55D
Bert (PI 557010)
M92-220
MN1410
MN1806CN
P2190C
P2358C
PI083945_3
PI250844
PI251585
PI378680D
PI437499
PI437869
PI437953A
PI438028
PI518754
PI538398
PI578421
PI597427A
PI612717
PI612721A
PI612732
R07C12
R18C55D
Wm82
XMAS Tree
CH 2018
CH 2019

Fig S3: Logistic function was used to model the light interception in different genotypes

a

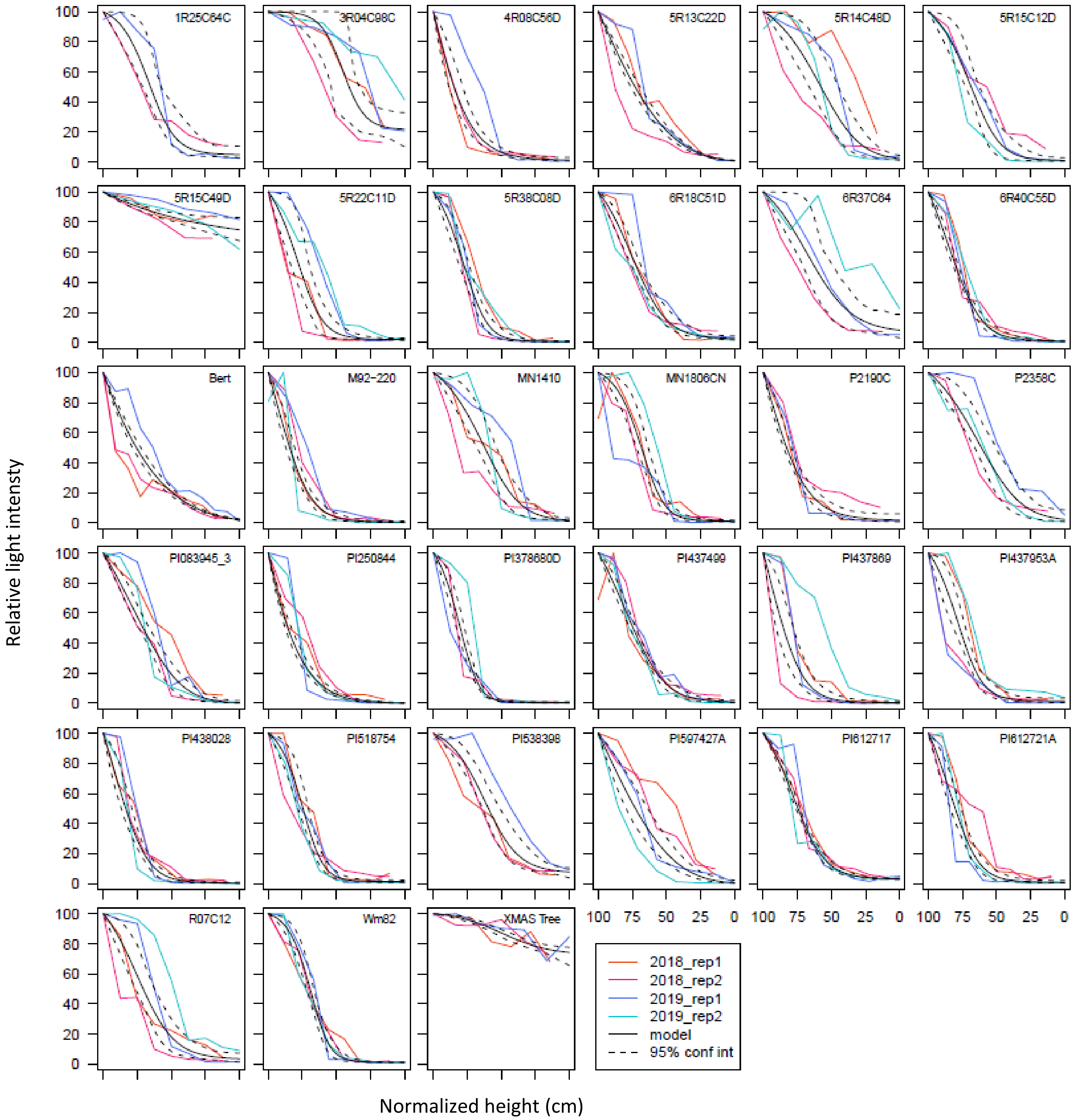

b

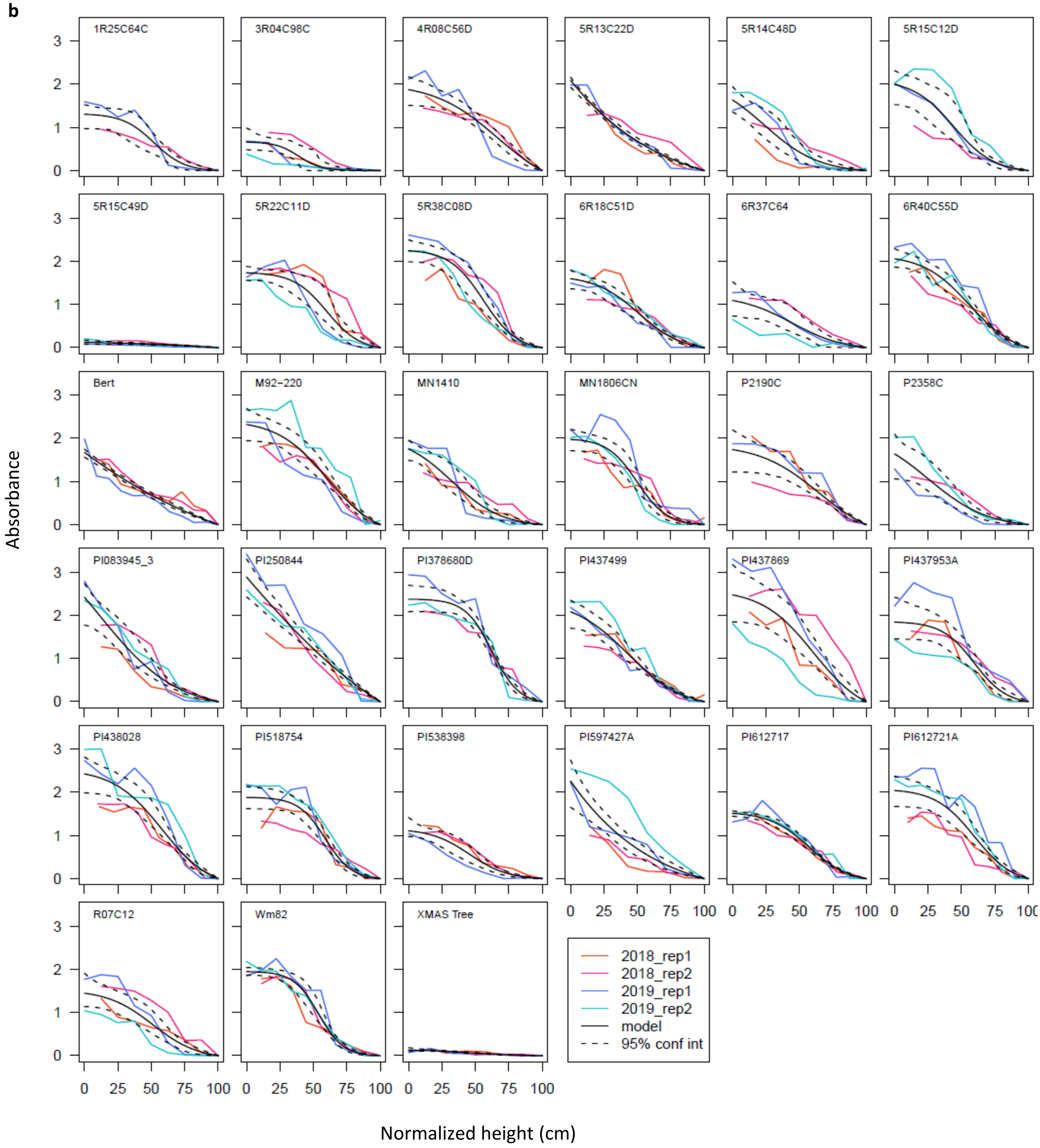

1R25C64C
3R04C98C
4R08C56D
5R13C22D
5R14C48D
5R15C12D
5R15C49D
5R22C11D
5R38C08D
6R18C51D
6R37C64
6R40C55D
Bert
M92−220
MN1410
MN1806CN
P2190C
P2358C
PI083945_3
PI250844
PI378680D
PI437499
PI437869
PI437953A
PI438028
PI518754
PI538398
PI597427A
PI612717
PI612721A
R07C12
Wm82
XMAS Tree
Absorbance
0
1
2
3
0
25
50
75
100
2018_rep1
2018_rep2
2019_rep1
2019_rep2
model
95% conf int
Normalized height (cm)

Fig S4 Beta distribution function was used to express the shape of soybean plants

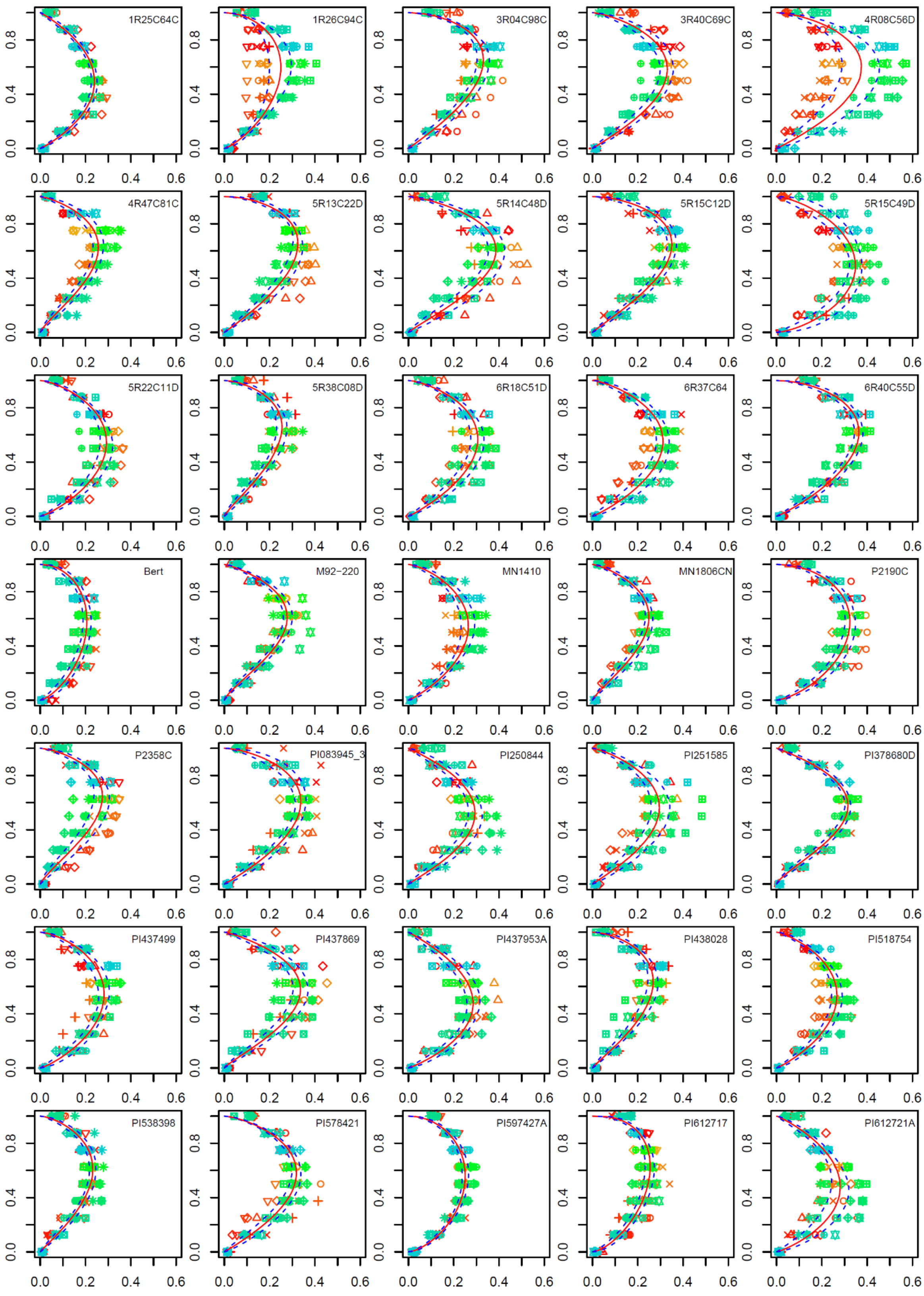

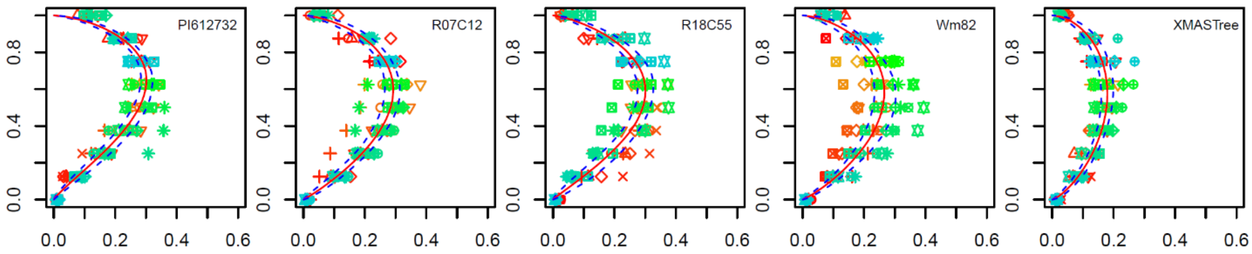

Fig S5: A simplified version showing the assumptions made for Co² assimilation rate estimation procedure

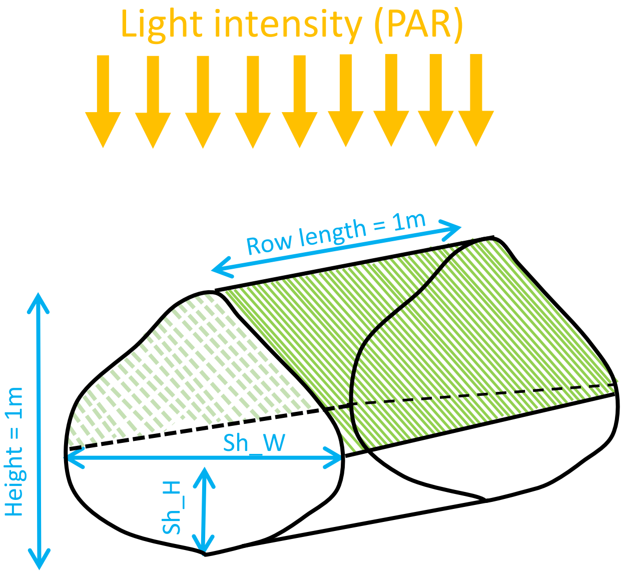

fig S6: Length of internodes and petioles from the top 4 nodes on the main stem of select genotypes

a

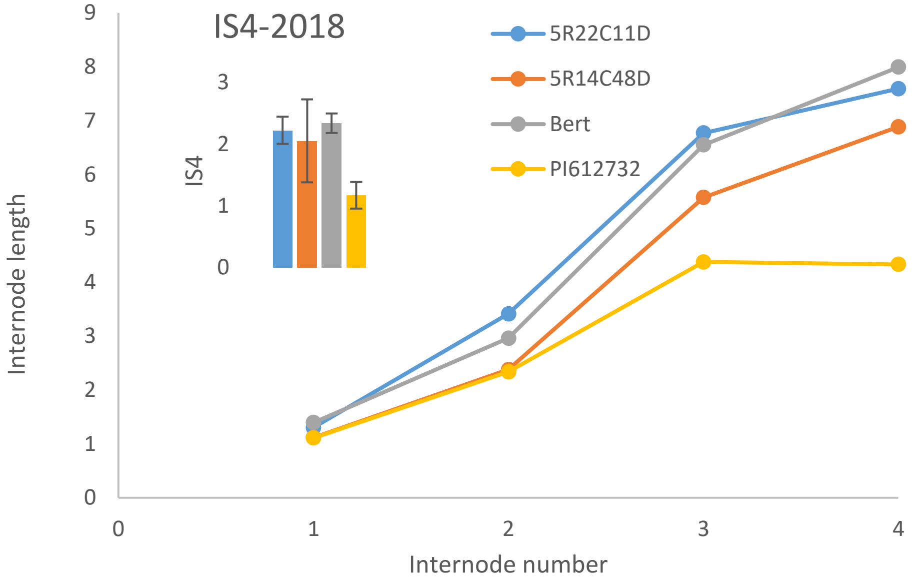

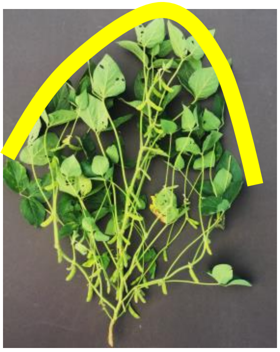

5R22C11D

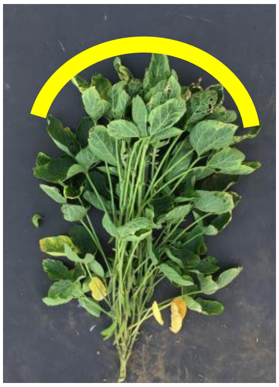

PI612732

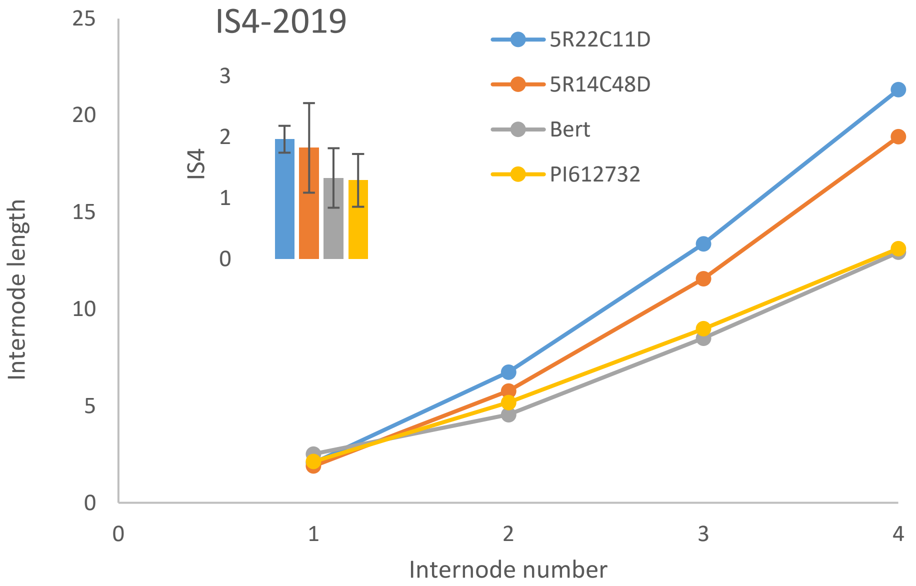

b

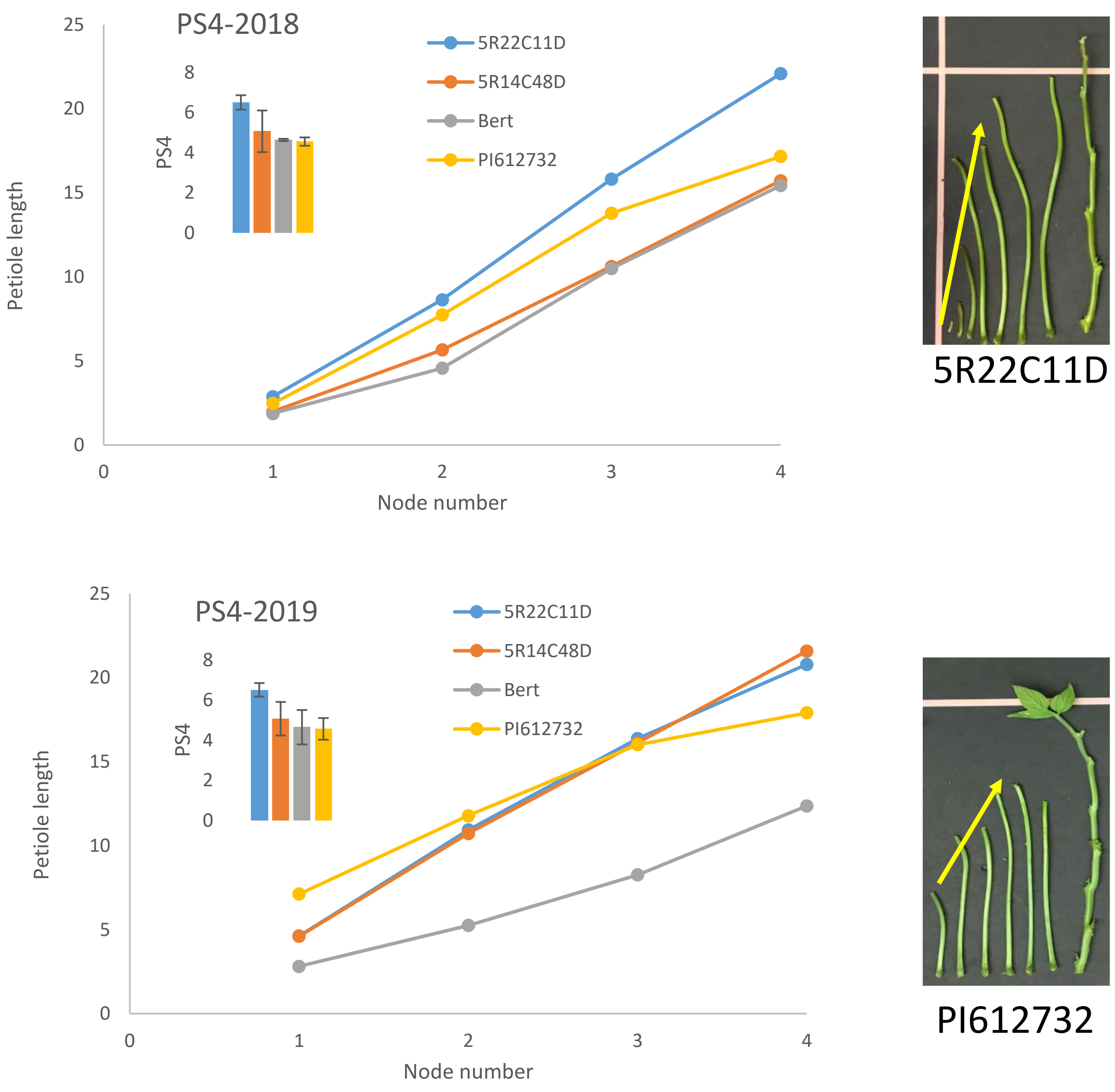

PS4-2018
Petiole length
25
20
15
10
5
0
Node number
0
1
2
3
4
PS4
8
6
4
2
0
5R22C11D
5R14C48D
Bert
PI612732
5R22C11D
PS4-2019
Petiole length
25
20
15
10
5
0
Node number
0
1
2
3
4
PS4
8
6
4
2
0
5R22C11D
5R14C48D
Bert
PI612732
PI612732

Fig S7: Imaging for branch angle measurements

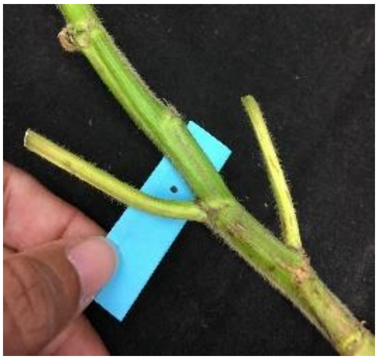

Supplement: Supplementary file 1 — Supplementary Material 1: Fig S1: Canopy coverage of all the accessions in the study over the planting season in 2018 and 2019 modeled by logistic regression. All the accessions included in the study are represented as individual logistic regression lines. Fig S2: Variation between different genotypes in all traits measured in the study in years 2018 and 2019. Averages for each trait was calculated at plot level in 2018 and 2019 and plotted as bar graph. Traits shown are canopy coverage (CC) traits: average canopy coverage (ACC), days to 50% canopy coverage (CC50), canopy coverage at R2 (CCR2), max growth rate (%/week) (MCC_w) and max growth rate (%/day) (MCC_d); light interception (LI) traits: photosynthetically active radiation at 50 % plant height (PAR50H); plant height at 50% photosynthetically active radiation (H50PAR) and photosynthetically active radiation at Ground (PARG); plant shape parameters: maximum height normalized (Sh_H) , maximum width relative to height (Sh_W) and area under the curve (Sh_A); shoot architecture traits: node number (NO), branch number (BN), branching zone (BZ), branching ratio (BR), branch angle (BA), branching density (BD), branching orientation (BO), leaf length (LL), leaf width (LW), leaf area (LA), petiole length at node 4 (PL4), petiole slope at node 4 (PS4), petiole angle node 4 (PA4), internode length at node 4 (IL4) and internode slope at node 4 (IS4). Error bars are standard deviations from mean. Fig S3: Logistic function was used to model the light interception in different accessions: (a) The PAR values shown as relative light intensity measured along every 10 cm increment from bottom to top the plants in a row (Y axis) was plotted against the height of plant, normalized to 1 m height to account for variation in height between accessions (X axis). Logistic fit for each rep (different colored lines) as well as the mean fit (black solid line) are shown for each accession. 95% confidence interval for the fit is indicated (bl [file 12870_2024_4859_MOESM1_ESM.pdf]
