# Supplementary material for: Variation in shoot architecture traits and their relationship to canopy coverage and light interception in soybean (Glycine max)
Source: BMC Plant Biol. 2024 Mar 16;24:194. doi: 10.1186/s12870-024-04859-2 (PMC10944616; doi:10.1186/s12870-024-04859-2)
Supplement: Supplementary file 8 — Supplementary Material 8 [file 12870_2024_4859_MOESM8_ESM.docx]

**Supplementary Methods S1: Additional information on plant shape analysis, estimating light interception at different heights along the canopy, estimating CO_2_ assimilation rate using the light interception values and canopy coverage estimation.**

**Plant shape analysis**

To automate plant shape outlining, first, we assumed that the plant outline shape is well-approximated by a rotation-symmetric surface in respect to the center line perpendicular to the ground surface. Second, we set the *x*-axis along this center line with *x* = 0 at the ground level and *x* > 0 for above the ground. Third, we set the *y*-axis perpendicular to the *x*-axis, so that the *y* value represents the distance between the outline and the center line (y ≥ 0). Fourth, since we consider geometrically similar shapes as the same shape, the distance from the center line value and the corresponding height value were scaled by the plant height. In this way, the outline shape of a plant is described as a unimodal curve that connects (0, 0) and (1, 0).

To parameterize the outline shape in this coordinate system, we used the probability density function of the beta-distribution ($f_{b}\left( x;\alpha,\beta\right)=\frac{1}{B\left( \alpha,\beta\right)}x^{\alpha-1}\left( 1-x \right)^{\beta-1}$, where $B\left( \alpha,\beta\right)=\int_{0}^{1} x^{\alpha-1}\left( 1-x \right)^{\beta-1}dx$; $0\leq x\leq1, \alpha>1,\beta>1$) because it can generate various smooth unimodal curves connecting (0, 0) and (1, 0) with varying values of two parameters. To make the parameters associated with the beta distribution interpretable, we re-parameterized the function as follows. By including the scaling factor for the distance, we used three parameters to describe the plant outline shape, the maximum distance from the center line *d_max_* (this is the distance scaling factor), the height for the maximum distance *h_dm_*, and the shape parameter *γ*. The $f_{b}\left( x;\alpha,\beta\right)$ was divided by its maximum value to make the maximum value 1 to obtain the max-value normalized beta-distribution $f_{nb}\left( x;\alpha,\beta\right)=f_{b}\left( x;\alpha,\beta\right)/f_{b}\left( h_{dm};\alpha,\beta\right)$, where $h_{dm}=\frac{\alpha-1}{\alpha+\beta-2}$. $\beta$ can be expressed by *h_dm_* and $\alpha:$ $\beta=\frac{\alpha-1}{h_{dm}}-\alpha+2$. Thus, the function can be reparameterized: $f_{nb}\left( x;\alpha,\beta\right)=f_{nbr}\left( x;\alpha,h_{dm} \right)$. Now the following non-linear model was fit using least square to eight observed distance values *d* and their corresponding relative height values *x* = 0.125, 0.25, 0.375, 0.5, 0.625, 0.75, 0.875, 1 to estimate the parameter values $d_{max},h_{dm},\alpha$ for each individual plant: $d\sim d_{max}f_{nbr}\left( x;\alpha,h_{dm} \right)$. The nlsLM function in the minpack.lm package was used for the non-linear least square fitting. Then the fitted values of the plants of each accession was refit with the model to obtain the accession specific $d_{max},h_{dm},\alpha$ values. The 95% confidence interval for each accession shown in (Fig **3a**; **S2**) was calculated by bootstrapping the plants of the accession 500 times. The shape parameter *γ* was defined as the area under the curve of $f_{nbr}\left( x;\alpha,h_{dm} \right)$; i.e., $\gamma=\int_{0}^{1} f_{nbr}\left( x;\alpha,h_{dm} \right)dx=1/f_{b}\left( h_{dm};\alpha,\frac{\alpha-1}{h_{dm}}-\alpha+2 \right)$. In this way, $\gamma$ was calculated from $\alpha$ and $h_{dm}$ as needed.

In summary, we described the plant outline shape by three parameters: the plant height, the maximum distance from the center line (Sh_W) is $d_{max}$, the height for the maximum distance(Sh_H) is $h_{dm}$, and the area under the curve (Sh_A)is γ.

**Estimating light interception at different heights along the canopy**

The PAR data were converted to the absorbance, -log­_10_(PAR at a particular height/PAR at the highest height), because the absorbance can be expected to be proportional to the height of the plant when we assume the geometric similarity and because the absorbance is independent of the PAR value at the highest height (note that the PAR value at the highest height could vary in actual PAR measurements). The absorbance values for each PAR measure (the same plant rows with varying heights) were normalized to 1 meter plant height as our interest in this analysis was the effect of the plant shape separate from the size factor, since there is a large variation for plant height in our study. We used accessions that had reasonably reproducible absorbance-vs-normalized-height relationships in at least one replicate in each of the years 2018 and 2019. The logistic model that goes through the point with absorbance = 0 and height = 1 (meter), $A=\frac{L}{1+e^{-k\left( x-x_{0} \right)}}-\frac{L}{1+e^{-k\left( 1-x_{0} \right)}}$, where $A$ and $x$ are the absorbance and the height, respectively, and $k<0$, was fit to the absorbance-vs-normalized-height data for each of the selected accession. Bootstrapping was used to estimate the mean and its confidence intervals of the model (Fig S2a and b).

**Estimating CO_2_ assimilation rate using the light interception values**

We estimated the CO_2_ assimilation rate (photosynthetic rate) for a one-meter-long plant row for each accession with the normalized plant height of 1 meter under different levels of PAR, using the beta-distribution-based plant outline shape model, the absorbance-vs-height model, and soybean light response curves. Using the soybean light response curves ( Zhang et al., 2011) we estimated the assimilation rates for each accession. The light response model was obtained by fitting an exponential decay with an asymptote line with a non-zero slope, $W=-e^{ap+b}+c+dp$, where $W$ and $p$ are the assimilation rate and PAR, respectively, and $a<0,d>0$, to the data that were manually obtained from the data points in Fig S3a. To simplify the assimilation rate estimation procedure, we used the following assumptions (Fig S4): (i) the shape of the plant row can be approximated by a prism with the cross section outline of the outline shape model based on beta distribution; (ii) the top-facing surface of the prism represents the photosynthetically active tissue; (iii) the light comes from straight up; (iv) however, the light intensity the surface receives is attenuated for a lower part of the plant according to the absorbance-vs-height model. Note that the actual PAR level that a particular part of the top surface receives is determined by (iv) and the angle of the surface to the direction of the light. To calculate the assimilation rate of the accession, first, the function to calculate the assimilation rate for a very narrow strip (the width of the strip was defined by height and delta height) of the top-facing surface along the 1-meter length of the plant row prism according to the normalized height of the strip was derived based on the PAR at the top of the plant row, the absorbance at the height, the angle of the strip surface relative to the light, and the light response curve. Second, the assimilation rate for the entire top-facing surface was calculated by integrating the function for the entire normalized height of the top-facing surface. Accessions with less reproducible absorbance data and extremely tall or short accessions were excluded from the absorbance-vs-height models.

**Canopy coverage estimation**

An unmanned aircraft system (UAS), DJI Inpsire 1, was used in this study to quantify CC.  The UAS was flown at an altitude of 70m with 70% overlap of images using the automated flight software of Pix4D capture (Pix4D S.A, Prilly, Switzerland).  Image orthomosaics were generated using Pix4D Desktop (Pix4D, SA, Prilly, Switzerland).  The WGS 84 datum was used with a projected coordinate system of UTM zone 15N.  The default processing options template, “Ag RGB,” was used for generating orthomosaics.   Nine ground control points were input into the Pix4D project directly following the initial processing step using the ray cloud editor.  Orthomosaics were loaded into Erdas Imagine (Hexagon Geospatial, United States) and unsupervised classification using k-means clustering into five classes was done on an indexed map of the ratio of red and green (R-G)/(R+G).  After unsupervised classification, the five classes were manually grouped into the “plant” class and the “soil” class based on interpretation of the five classes.  The classes were recoded, and a mask was set based on the new classification. Data was extracted using QGIS software (QGIS Geographic Information System. Open Source Geospatial Foundation Project. http://qgis.osgeo.org).  In QGIS, a shape file was created where field plot polygons were used to identify each of the plots.  The zonal statistics plugin was used to extract the CC as the proportion of “plant” class to the entire plot (“plant” + “soil”).
